# Summertime Primary and Secondary Contributions to Southern Ocean Cloud Condensation Nuclei

**DOI:** 10.1038/s41598-018-32047-4

**Published:** 2018-09-14

**Authors:** Kirsten N. Fossum, Jurgita Ovadnevaite, Darius Ceburnis, Manuel Dall’Osto, Salvatore Marullo, Marco Bellacicco, Rafel Simó, Dantong Liu, Michael Flynn, Andreas Zuend, Colin O’Dowd

**Affiliations:** 1 School of Physics, Ryan Institute’s Centre for Climate & Air Pollution Studies, and Marine Renewable Energy Ireland, National University of Ireland Galway,University Road, Galway, H91 CF50 Ireland; 2 Institut de Ciències del Mar (CSIC), Barcelona, Catalonia Spain; 3 Agenzia nazionale per le nuove tecnologie, l’energia e lo sviluppo economico sostenibile, ENEA — Centro Ricerche Frascati, Frascati, Italy; 4 Institute of Atmospheric Sciences and Climate (ISAC), Rome, Italy; 5 Sorbonne Université, CNRS, Laboratoire d’Océanographie de Villefranche, LOV, F-06230 Villefranche-sur-Mer, France; 6 Centre for Atmospheric Sciences, School of Earth and Environmental Sciences, University of Manchester, Manchester, M13 9PL UK; 7 Department of Atmospheric and Oceanic Sciences, McGill University, Montreal, Quebec, Canada

**Keywords:** Atmospheric dynamics, Atmospheric dynamics, Ocean sciences, Ocean sciences

## Abstract

Atmospheric aerosols in clean remote oceanic regions contribute significantly to the global albedo through the formation of haze and cloud layers; however, the relative importance of ‘primary’ wind-produced sea-spray over secondary (gas-to-particle conversion) sulphate in forming marine clouds remains unclear. Here we report on marine aerosols (PM_1_) over the Southern Ocean around Antarctica, in terms of their physical, chemical, and cloud droplet activation properties. Two predominant pristine air masses and aerosol populations were encountered: modified continental Antarctic (*cAA*) comprising predominantly sulphate with minimal sea-salt contribution and maritime Polar (*mP*) comprising sulphate plus sea-salt. We estimate that in *cAA* air, 75% of the CCN are activated into cloud droplets while in *mP* air, 37% are activated into droplets, for corresponding peak supersaturation ranges of 0.37–0.45% and 0.19–0.31%, respectively. When realistic marine boundary layer cloud supersaturations are considered (e.g. ~0.2–0.3%), sea-salt CCN contributed 2–13% of the activated nuclei in the *cAA* air and 8–51% for the marine air for surface-level wind speed < 16 m s^−1^. At higher wind speeds, primary marine aerosol can even contribute up to 100% of the activated CCN, for corresponding peak supersaturations as high as 0.32%.

## Introduction

The Earth’s radiative budget encapsulates the difference between incoming solar shortwave radiation, less its reflected and back-scattered component plus a longwave (absorbed and re-emitted) radiation component, effectively determining the global climate. It is strongly influenced by the marine boundary layer (MBL) as ocean surfaces are darker and more absorbing on average than land masses, and with 70% share of the Earth’s surface. This dark ‘bottom’ of the MBL is often covered by marine aerosol haze and cloud layers which, even if optically thin, have a strong reflectance effect due to the dark underlying surface. The contribution of marine aerosol to the radiative budget is both direct^1^, through aerosol scattering and absorption, and indirect^2^, through cloud droplet activation and subsequent influence on cloud radiative properties. The indirect effect is mainly determined by the availability and physico-chemical properties of the marine cloud condensation nuclei (CCN) population, a subset of the marine aerosol.

There are two generic types of marine aerosol, primary (PMA) and secondary (SMA), both with differing impacts on the direct and indirect aerosol radiative effect. PMA is predominantly comprised of sea-salt with varying fractions of organics^3^; while SMA is generally comprised of non-sea-salt sulphate (nss-SO_4_), whose marine precursor is ocean-released dimethylsulphide (DMS). DMS is a volatile by-product of the planktonic food web formed by enzymatic degradation of the abundant phytoplankton osmolyte dimethylsulphoniopropionate^4^. Atmospheric DMS oxidation mainly leads to nss-SO_4_^5^ and, in second place, methanesulphonic acid (MSA). SMA can often be an internal mixture of nss-SO_4_ with ammonium, MSA, organics, or amines^6^.

Both PMA and SMA have been proposed as central to various atmospheric climate responses to counteract greenhouse gas radiative forcing helping to stabilise climate change: for SMA, this involves the marine sulphur cycle, CCN, and clouds while for PMA, this involves wind speed, CCN and clouds^7,8^. The proposed aerosol response is typically based on an increase in CCN in a warmer climate, which has been observed^9,10^, leading to an increase in cloud droplet concentration and related reflectivity of low-level clouds (a net radiative cooling effect). However, it is still unknown whether environmental changes from increased cooling (or from other environmental factors such as increasing atmospheric CO_2_ concentrations) will incur positive or negative responses in DMS production, due to the complexity of its generation in nature^11^.

Organic matter (OM) enrichment in PMA might also have competing effects on radiative forcing due to dichotomous effects in water uptake. It has been shown that OM in PMA, possibly in the form of marine hydrogels, can increase the activation efficiency of sea-salt CCN in supersaturated ambient N.E. Atlantic conditions leading to high levels of cloud droplet number concentration (CDNC ~500 cm^−3^) in marine stratocumulus^12^. Yet, laboratory studies^13^ observed a hydrogel proxy leading to little enhancement of sea-salt activation but found that the salt significantly increased the activation potential of the OM. Other mesocosm experiments found negligible change in CCN concentration with bloom activity^14,15^.

Mesocosm experiments^16^ agreed somewhat better with the N.E. Atlantic ambient studies^17^ for sub-saturated air, illustrating reductions in scattering from various OM enrichments. However, there were still many differences between the bloom mesocosm studies and ambient air- for example, the former study found hygroscopicity followed volume mixing rules (i.e. the Zdanovskii-Stokes-Robinson (ZSR) mixing rules^18^), while the latter found “bistable” behaviour.

Also, great differences arise in the observed primary OM enrichment and its correlation to productivity proxies (e.g. chlorophyll-*a*). Simultaneous analysis of *in-situ* chlorophyll and artificially-generated sea-spray in (calm) N.E. Atlantic surface waters found very low and invariant OM mass fractions (around 0.15) in this sea-spray and also reported no correlation with chlorophyll^19^. This contrasts with the ambient studies over the N.E. Atlantic^20^ that reported a ‘biological’ time delay relating to bloom senescence where the correlation coefficient between chlorophyll and OM fraction increased from 0.67 (daily) to 0.85 (monthly). In agreement with the latter ambient studies^20^, a time lag was also clearly seen in the above mesocosm studies^16^. In addition to the reported effect of primary OM on water uptake properties, SMA has also been shown to host surfactants which can also lead to profound increases in CCN concentrations^21^.

It is still unclear why these differences arise in sea spray, and while variable experimental approaches likely hold some element of truth, the possibility of different characteristics associated with different oceanic waters also plays a role in the differing primary OM properties. A preliminary study^22^ into Southern Ocean aerosol to explore the link between productivity and cloud properties was successful in demonstrating a link between bloom biogenic isoprene emissions and CCN. More recently McCoy, *et al*.^23^, in linking satellite observations of cloud albedo to model assessment of sources, found enhanced *CDNC* is spatially correlated with regions of high chlorophyll-*a*, and the spatiotemporal variability in *CDNC* is found to be driven mostly by high concentrations of sulphate aerosol at lower Southern latitudes and by organic matter in sea spray aerosol at higher latitudes.

The Southern Ocean is a natural laboratory to study pristine marine aerosol production and sources due to its remoteness from anthropogenic activity as demonstrated in previous studies, both long-term and short-term aerosol measurement campaigns^24–26^. In this study, we analyse data gathered from a cruise around the Atlantic sector of the Southern Ocean from January to February 2015, to characterise aerosol physico-chemical properties associated with different source regions and regional air masses. Using a suite of *in-situ* instrumentation measuring physical and chemical properties of PM_1_ marine boundary layer aerosol, we characterise the key physico-chemical properties of the Southern Ocean aerosol and CCN population, and the contribution of PMA and SMA to the inferred activated, or actual, CCN.

## Results

### Regional air mass sources

**Figure 1:**
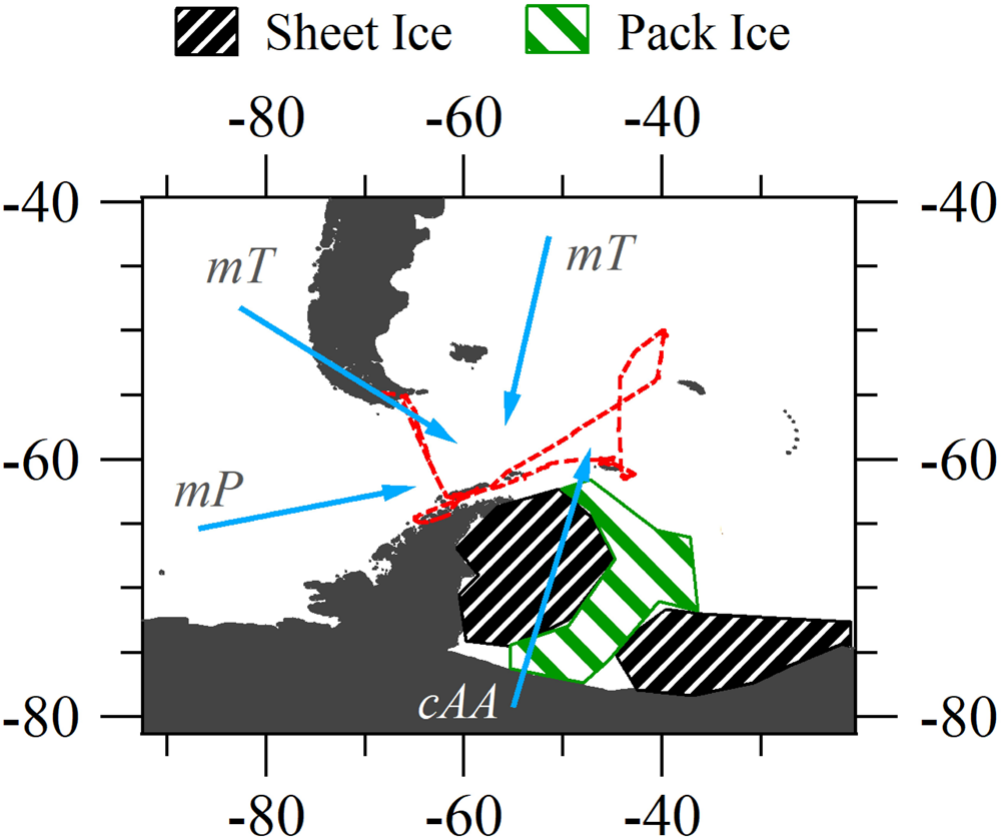
Map of Southern Ocean region, ship course marked in red. Movement of air masses from three principal source regions shown in blue arrows; continental Antarctic (*cAA*), maritime Polar (*mP*) from the West, and maritime tropical (*mT*) or modified-*mT*, both from north and northwest of −60° latitude. Approximate summer regions of pack ice (green striped) and lasting sheet ice (black striped) in the Weddell Sea are shown.

This study characterises steady-state aerosol properties associated with different air masses in the oceanic region around Antarctica, and in particular the Scotia Sea. Steady state conditions refer to aerosol microphysical properties varying less than 20% over a selected period of eight hours (although an odd exception was made for shorter periods - see further details in the Methods and Supplementary Material). Analysis of the data revealed 12 pseudo-steady state periods, shown in Figure S3, from which 2 main prominent air masses were identified and one less prominent, as shown in Fig. 1. The two predominant air masses were continental Antarctic (*cAA*) and maritime polar (*mP*), while the less frequent air mass was maritime tropical (*mT*) which was generally associated with polluted incursions from South American outflow.

### Physico-chemical aerosol properties in *cAA* air masses

The *cAA* air masses formed over Antarctica are amongst the coldest and driest in the world. During this cruise, *cAA* air masses encountered were sourced from the Antarctic plateaux flowing Northward in the katabatic outflowing wind driven by the subsiding free troposphere (FT) air in the high-pressure polar region above the plateaux. The *cAA* outflow traverses initially ice surfaces which evolve into broken pack ice as the air advects out over the Weddell Sea, *en route* to the open oceanic waters. In summer, as the pack ice replaces the winter sheet ice, the biologically-rich waters begin to open up, presumably enabling the sea-air flux of particulates and gasses. Our retrieved HYSPLIT^27^ trajectories reveal that over the Weddell Sea the air flow is generally associated with stagnant high-pressure systems taking, at times, up to 90–120 hours to penetrate through to the open waters. All the *cAA* periods discussed here show the highest chlorophyll-*a* (Chl-a) mean concentration air trajectory exposures (see Methods) and are likely influenced by biologically productive waters as the air advected over the Weddell Sea and, strictly speaking, are modified *cAA* air masses.

**Figure 2:**
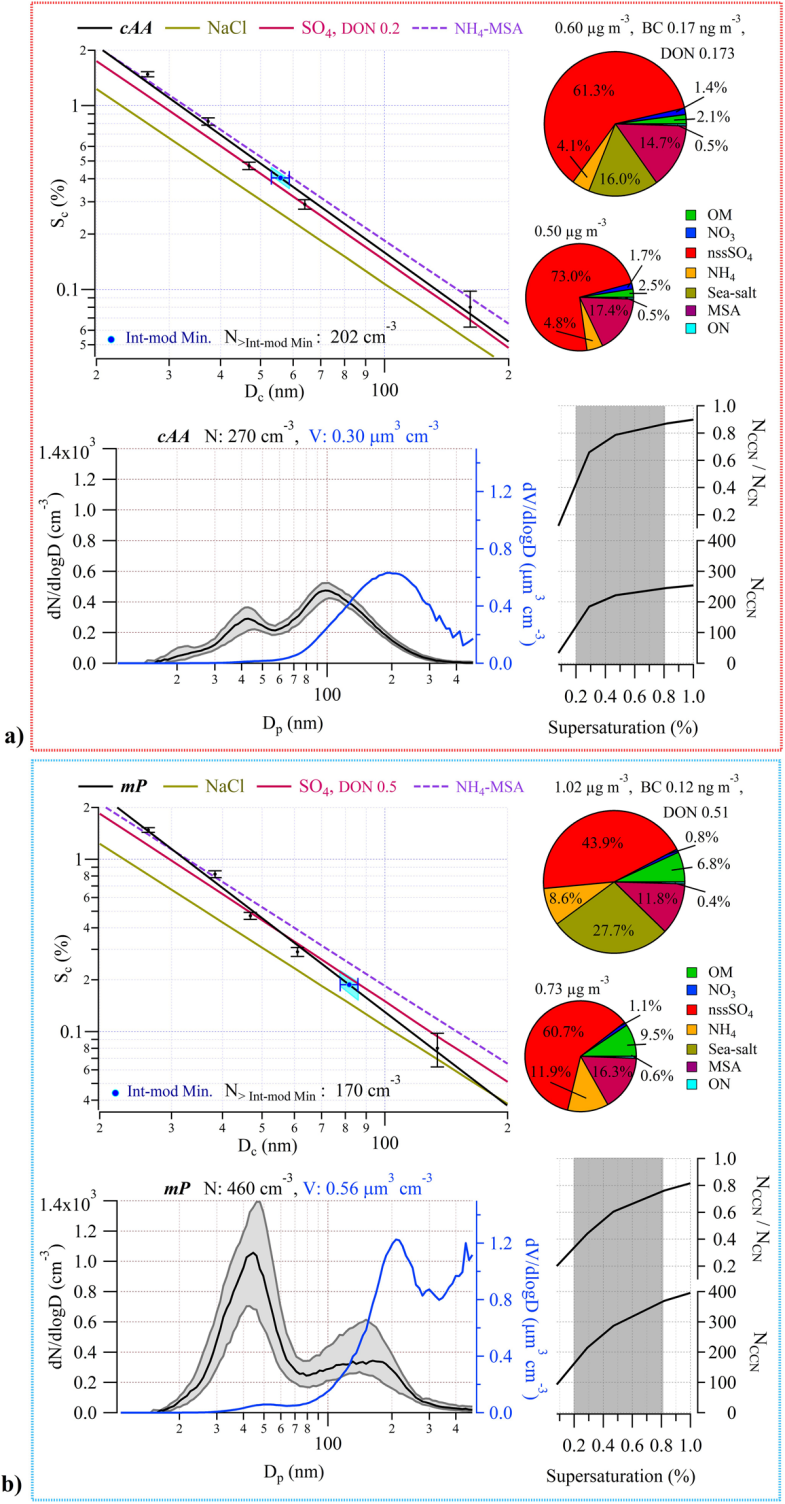
Physical and chemical properties of *cAA* (Weddell Sea influenced) air masses (**a**) and *mP* air masses (**b**). All data from each steady-state case are lumped together into an air mass average. Bottom left: Median number-size distribution (black) and volumetric size distribution (blue), with *D*_*p*_ the SMPS-derived dry particle diameter. Shaded grey area represents 25^th^–75^th^ percentile range with the total particle number and corresponding volume concentrations noted at the top. Bottom right: On top, the ratio of CCN to all particles greater than 20 nm, on bottom, the total number of CCN for varying supersaturation. Shaded range of supersaturations represent typical values for marine stratocumulus clouds. Top left: CCN activation efficiency as a function of critical supersaturation and particle diameter, on top, the Inter-modal minima point indicated in blue, with 10% SMPS sizing error (the blue shaded region corresponds to the spread of *S*_*c*_ values considering SMPS sizing errors) and the total number concentration of particles at sizes greater than the inter-modal minima. In black, fitted CCN activation curve obtained from the measurements with corresponding error bars. The red line represents partially neutralized sulphate according to the DON indicated (using mixture of H_2_SO_4_ + (NH_4_)_2_SO_4_), the olive brown line represents NaCl, and the purple dashed line represents ammonia-neutralised MSA (i.e. NH_4_-MSA salt) where all lines are based on predictions by the AIOMFAC model. Top right: Pie chart of chemical mass fractions, with the total mass concentration, average black carbon concentration, and DON_mol_ noted on top. The smaller pie chart is the breakdown of the non-sea-salt aerosol species.

Each air mass hosted aerosol with distinctive characteristics associated with that particular air mass, therefore both the *cAA* cases and *mP* cases were each consolidated, respectively, into a representative air mass type ‘average’ for ease of comparison, shown in Fig. 2. The individual periods included in the consolidated dataset are displayed in Figure S3, where the *cAA* case is combined *cAA* periods 1, 2, 3, and 4, and where the *mP* case is combined *mP* periods 1, 3, 4, and 5).

For the *cAA* case average, the total aerosol and CCN concentrations (at the highest measured supersaturation of 0.8%) were 270 ± 40 cm^−3^ and 217 ± 31 cm^−3^, respectively. The submicron size distribution was bimodal, exhibiting a log-normal Aitken mode distribution with modal diameter peaking at 42 nm and a log-normal accumulation mode distribution with modal diameter peaking slightly larger than 100 nm. The latter mode contributed 70% of the submicron number concentration. When the bimodal distribution is fitted with log-normal modes, the inter-modal minimum can be accurately calculated to be 56 nm (see Table S1). The *cAA* air mass average mass concentration was 0.60 ± 0.16 µg m^−3^, with black carbon concentrations of 0.17 ng m^−3^ confirming the pristine nature of the air, and DON_mol_ of 0.17 pointing to a quite acidic aerosol. Sea-salt mass fraction derived from the AMS reveals a very modest 16% contribution (0.095 µg m^−3^) to the total PM_1_ mass, and even smaller contribution from OM (2%). The majority of the chemical composition is comprised of nss-SO_4_ (61% by mass), ammonium (4%), and MSA (15%).

To explore the cloud droplet nucleating, or CCN activation, ability, we deployed an experimental set up (see Methods) to enable us to produce CCN activation lines (i.e. plots of the critical supersaturation, *S*_*c*_, versus the critical dry diameter, *D*_*c*_) for both lab and environmental scenarios. In measuring the water uptake properties of the aerosol, this powerful setup elucidates many important properties of the CCN population through comparative analysis with modelled particle hygroscopicity, particularly the inferred composition of the nuclei as a function of size. The associated critical diameter curve (i.e. *S*_*c*_ v *D*_*c*_), or activation lines, for *cAA* aerosol indicates a chemically homogeneous aerosol as a function of size but with activation efficiency slightly less than ammonium sulphate (i.e. there is a 12.2% deviation in the *S*_*c*_ for the environmental sample compared to that theoretically calculated for partially neutralised sulphuric acid at 27 nm, and a 7.1% deviation at 105 nm). The activation lines depend on the hygroscopicity parameter *κ*, which in our case is derived from CCN activation measurements; however, CCN-derived *κ*-values display a variance dependent on particle size (the Kelvin effect) whereby smaller Aitken mode particle sizes show the largest *κ* variance. This variance arises in *κ*-Köhler theory, as *κ* is associated with the water activity of a droplet rather than its size, because this study uses sophisticated water activity vs. composition resolved values based on the Aerosol Inorganic-Organic Mixtures Functional groups Activity Coefficients (AIOMFAC) model (see Methods). This can be seen for a species like ammonium sulphate for which *κ*-values differ from the accepted value of 0.61^28^ with size-dependence^21^. For the *cAA* activation efficiency curve, small differences between *D*_*c*_ show variations in *κ* (7.1% difference in *κ* at 26.8 nm up to 13.6% difference of *κ* at 104.2 nm, see Table 1) which are not within the bounds of the expected variations and point to some slight chemical inhomogeneity in the measured size range.

**Table 1:** Log-normal fit parameters for number and volume size distributions.

	N		*Κ*	AS_eq_ *κ*	V	
	Peak, nm	*δ*			Peak, nm	*δ*
***cAA***	26.8	1.28	0.41	0.44	—	—
	*41.5*	*1.20*	*0.45*	*0.57*	—	—
	104.2	1.53	0.55	0.63	193.0	1.60
***mP***	42.2	1.32	0.47	0.57	—	—
	138.7	1.52	0.99	0.64	212.1	1.38
	—	—	—	—	483.3	1.29

Particle dry diameter (peak diameter, nm), spread (*δ*), *κ*, and equivalent *κ*-value for pure ammonium sulphate at that particle size (AS_eq_ *κ*) for fitted number peaks shown on left. Particle volume-size distribution (peak diameter, nm) and spread (*δ*) of the fitted peaks shown on right. Empty spaces indicate that corresponding peaks have an amplitude too small to be fitted reliably. All *κ* computed assuming a constant surface tension of 0.072 N m^−1^.

On the *S*_*c*_*-D*_*c*_ plot (Fig. 2a), the inter-modal minimum (taken from the aerosol size-distribution and thought to represent the average critical activation diameter for the activation of the size distribution into ambient clouds) occurs at a critical supersaturation of ~0.40%. Using the theoretically derived activation curves of partially neutralised sulphate as a comparison, the inter-modal minimum occurs at a *D*_*c*_ on the measured environmental activation curve well above the corresponding *D*_*c*_ on the theoretically derived curve. This means that the *S*_*c*_ at the inter-modal minimum of the measured aerosol is larger than the *S*_*c*_ of aerosol composed entirely of partially or completely neutralised sulphate at that same size. This is a reduction of activation potential from that of ammonium sulphate particles. As this is in opposition to the increase in activation potentials that would be affected by sea-salt and/or (NH_4_)_2_SO_4_ - H_2_SO_4_ aerosol, the reduction is instead likely due to a mixture of (water-soluble) organics and NH_4_ neutralising MSA (see Figure S1). This analysis (see Methods) suggests little contribution of primary sea-spray aerosol to ambient *cAA* CCN abundance.

When a cloud forms on an initial monomodal distribution (or an existing bimodal distribution),the activated droplets selectively grow in (dry) size as the droplets act as dilute aqueous chemical reactors converting SO_2_ into sulphate^29^. In a bimodal number-size distribution where this process has occurred, the inter-modal minimum can be regarded as the average aerosol sample- *D*_*c*_. Other growth processes leading to bimodality, such as coalescence and Brownian scavenging, are not considered here following arguments made by O’Dowd, *et al*.^30^, due to the cloudiness of the region (Figures S6 and S7), low particle concentration, and the large sized diameter of the modes. Essentially, during cloud droplet formation, a ‘bite’ is taken out of the large-diameter side of the monomodal aerosol distribution and these are activated nuclei that grow in nuclei mass *via* aqueous phase sulphate production in the cloud. On exit from the cloud, the emerging aerosol is bimodal, with the activated nuclei forming a larger-diameter (accumulation) mode. Hence all particles larger than *D*_*c*_ are regarded as having been activated into cloud droplets previously and all particles smaller than *D*_*c*_ are non-activated interstitial aerosol and remain as an Aitken mode. Using this approach, we can evaluate from our measurements that, for the average of *cAA* aerosol forming a cloud, the *D*_*c*_ is 56 nm, resulting from a peak supersaturation of *S*_*c*_ ≈ 0.4%, leading to the activation of 202 droplets cm^−3^ from a total aerosol population of 270 cm^−3^. Combining this approach with the activation line analysis (see Methods), we calculate that for the range of peak supersaturations (0.34–0.45%) observed in the *cAA* cases, 2–13% of the activated CCN are PMA (see Table 2).

**Table 2:** Values used for PMA number contribution calculation.

	*S*_*peak*_, %	*D*_*SMA*,_ *nm*	*D*_*PMA*,_ *nm*	*act SMA, cm* ^−3^	*act PMA, cm* ^−3^	% *PMA*
*cAA 1*	0.41	50.7	41.3	185	27	12.7
*cAA 2*	0.35	58.6	45	150	15	9.1
*cAA 3*	0.45	48.4	38.2	205	4	1.9
*cAA 4*	0.34	59.9	45.9	170	6	3.4
*mP 1*	—	—	—	—	—	—
*mP 2*	0.31	61	48.9	203	18	8.1
*mP 3*	0.2	81.8	64.8	32	33	50.5
*mP 4*	—	—	—	—	—	—
*mP 5*	0.19	85	66.3	216	47	17.8
*Case cAA*	0.40	51.8	41.7	175	25	12.4
*Case mP*	0.19	86.8	69.3	130	39	23.0
*E1*	0.30	63.8	50.4	63	102	61.8
*E4*	0.32	61.8	48.8	0	62	100.0

Inter-modal minimum (D_c_) values gave corresponding cloud peak supersaturation (S_peak_) based on ambient S_c_-D_c_ curves. The same curves resolved the critical diameters of SMA (D_SMA_) and PMA (D_PMA_) which were used in conjunction with number-size distribution data to find the number of activated SMA and PMA particles and find the percent contribution of PMA (% PMA) to the cloud droplet number. The number concentration of PMA and SMA has an associated ±5 cm^−3^.

### Physico-chemical aerosol properties in *mP* air masses

The *mP* air masses encountered formed over cold unfrozen polar marine waters, around 60 °S latitude. These air masses are moist but constrained in terms of total water content by the cold environment. During Antarctic summer, the polar regions above ~65 °S latitude are characterised by prevailing easterlies near the ocean/land surface, while at mid-latitudes (30 °S–60 °S) westerlies prevail (see, e.g., observational data and model predictions by Broeke, *et al*.^31^). The *mP* air masses during the cruise typically advected with the westerly prevailing winds as part of the Circumpolar Antarctic Circulation. The *mP* air masses sometimes return south as returning-*mP* air masses following short excursions to the north. From the selected periods in this study, all *mP* trajectories are consistently open ocean trajectories even though some may have advected relatively close to, or over, coastal waters.

By way of contrast to the *cAA* aerosol, the *mP* aerosol had an average total number concentration of 460 ± 223 cm^−3^ (i.e. double that in the continental air) while activated CCN at the highest measured supersaturation (0.8%) was 420 ± 168 cm^−3^. The marine aerosol possessed a dominant Aitken mode at about 42 nm dry mobility diameter, but in contrast to the *cAA* aerosol had an amplitude four times that of the accumulation mode, which peaked at 140 nm. The inter-modal minimum was found at 82 nm, corresponding to critical supersaturation of 0.19% on the *S*_*c*_*-D*_*c*_ CCN activation curve (Fig. 2b). It is important to note that the Aitken mode at approximately 42 nm was remarkably stable regardless of variation in modal amplitude or accumulation mode peak diameter. Also, in sharp contrast to the *cAA* aerosol, total mass was 1.02 ± 0.45 µg m^−3^ (compared to ~0.60 µg m^−3^ in the *cAA* case) and the volumetric size distribution peaked at 210 nm and 480 nm, although the larger peak was fit with available data and may in fact peak at a larger diameter. The *mP* air mass had an average black carbon mass concentration of 0.12 ng m^−3^, also indicating pristine air, however DON_mol_ was 0.51 suggesting more neutralised aerosol. More sea-salt, not surprisingly, was evident in the marine aerosol with the AMS-derived mass concentrations showing a total mass concentration of 0.282 µg m^−3^, of which 28% was sea-salt. Chemical contributions in the *mP* aerosol were otherwise dominated by nss-SO_4_ (44%), MSA (12%), ammonium (7%), and OM (7%). The *mP* air masses at 0.3% supersaturation activated ~200 CCN cm^−3^ and at 0.8% supersaturation approximately 400 CCN cm^−3^ were activated, changing the N_CCN_/N_CN_ ratio from 0.4 to 0.7. The ratios are lower than the equivalent *cAA* aerosol, pointing to a lower fraction of the aerosol activating in the *mP* air.

In the critical activation curve (Fig. 2b), the slope is characteristic of a size-varying chemical composition for the CCN, suggesting different CCN species activating at different supersaturations and critical diameters. This is seen as the activation line of the environmental sample deviates from a parallel slope associated with any of the different homogeneous chemical species or fixed compositions and diverges from the sulphate species line, with a DON_mol_ of 0.5, towards the NaCl activation line. The measured ambient critical CCN activation curve shows an inferred 23% contribution of primary sources at the inter-modal minimum. For the larger nuclei around 55 nm < *D*_*c*_ < 200 nm, *S*_*c*_ lies between that of standard values for sea-salt and ammonium sulphate, with a corresponding hygroscopicity parameter at CCN activation of *κ* = 0.99 at 140 nm, while the activation of particles below 50 nm require a higher *S*_*c*_, *κ* = 0.47 at 42 nm (assuming a constant surface tension of 0.072 N m^−1^). For the average of *mP* aerosol, the *D*_*c*_ is 82 nm, corresponding to a peak supersaturation of *S*_*c*_ ~ 0.19%, leading to the probable activation of 170 cm^−3^ droplets from a total aerosol population of 460 cm^−3^. For the suite of *mP* cases, the relevant range of *S*_*c*_ was calculated as 0.19% to 0.31%, with an 8–51% PMA contribution to the activated CCN or cloud droplet population in *mP* air (see Table 2). The 51% case, *mP* 3, corresponds to a wind speed of 16 m s^−1^ which was the highest wind speed during any steady-state case sampled.

### Physico-chemical aerosol properties *mT* air masses

Maritime tropical (*mT*) air masses were least frequently observed, typically emerging from the South Atlantic (or Pacific) sub-tropical high-pressure region and at times, these air masses could briefly traverse South America lending to a modified (tropical) marine air mass which invariably becomes polluted from South American outflow. The observed *mT* incursions exhibited characteristics very like *mP* air masses, as both represent maritime conditions, except one period (*mT* 1) that showed a very low percentage of primary aerosol loading with an elevated black carbon concentration (30 ng m^−3^) and the largest overall particle concentration (1,165 cm^−3^). *mT* 1 can be classified as an *mT* anthropogenically modified air mass. The *mT* air mass total number concentrations varied from 550–640 cm^−3^, with little variation of CCN number concentrations at 0.8% supersaturation, excluding pollution-modified *mT* air masses (Figure S3). The two un-modified *mT* periods show number and volume size-distributions very similar to *mP* aerosol, with a dominant Aitken mode compared to the accumulation mode. However, the difference in the number concentration between Aitken and accumulation modes was less, being almost double as opposed to quadruple in *mP* periods. The only fully modified *mT* period also appeared as the only monomodal pseudo-steady state period observed. The *mT* air mass chemical composition was similar to *mP* periods, except with a smaller fraction of MSA and increased fraction of OM. Generally, the BC concentration is higher than what is considered pristine for the *mP* air masses in the Scotia Sea region (<0.2 ng m^−3^) but still clean (<0.5 ng m^−3^) for marine aerosol. The N_CCN_/N_CN_ ratio revealed distinct fractions for 0.3% supersaturation ranging from 0.3 to 0.6, but reached 1 at 0.8% supersaturation (Figure S2). The *mT* air masses show similar log-scale slopes on the *S*_*c*_*-D*_*c*_ curves to *mP* air masses in terms of activation efficiency (see Figure S3), but *mT* 1 followed an activation efficiency like neutralised sulphate and had increased activated fractions like *cAA* aerosol (see Figure S2), which is a typical behaviour for such low concentrations of sea-salt (~0.02 µg m^−3^).

## Discussion and Conclusion

In contrast to other oceanic waters (e.g. the North Atlantic), the continental air outflowing from the polar region after subsiding from the free troposphere, and even becoming modified with marine sources as it advects over broken pack-ice, has brought with it a quite low-complexity aerosol population, seemingly comprising almost exclusively of biogenic sulphate products (nss-SO_4_ and MSA, and their neutralised variants) and close to insignificant values of organic aerosol. Similarly, the *mP* air carrying the most ‘maritime’ aerosol comprises simply of similar sulphur species plus sea-salt. Other species (e.g. organic nitrogen, primary organic matter) were present in such minute quantities that their ability to influence atmospheric processes of note seems to be limited, if anything, to potential involvement in nucleation and cluster growth processes.

The concentration of CCN is typically reported over a wide supersaturation typically ranging from 0.1% to 1%^32–35^. On average, the number of CCN in *mP* air masses was effectively double the concentrations found in *cAA* air masses at a supersaturation of 0.8%. However, at a lower supersaturation of ~0.3%, *mP* air masses exhibited similar CCN abundance as *cAA* air masses, of approximately 200 CCN cm^−3^. In *mP* and *mT* aerosol, about 75% of the aerosol number population resided in the Aitken mode (which can be considered un-activated CCN) in contrast to *cAA* aerosol where 70% resided in the accumulation mode (which can be considered activated CCN)^29^.

As reported previously^36^, overall, the largest observed SMA component in all air masses was nss-SO_4_, and sea-salt dominated the PMA component. At low supersaturations of the order of 0.1–0.2%, sea-salt tended to dominate the activated fraction particularly under high winds. In *mP* air masses, the CCN activation efficiency closely follows that of pure sea-salt for the majority of particles with *D*_*p*_ > 200 nm, confirming that CCN particles at low supersaturation are principally PMA in origin. The fraction of CCN that are PMA decreases with increasing *S*_*c*_, gradually reducing to about 20% or less at the inter-modal minima below which CCN are almost entirely SMA; however, due to the size-differentiated composition of marine aerosols, this is to be expected. Nevertheless, our results suggest that average supersaturations larger than 0.45% are not relevant in this region.

Our results here are presented for steady state scenarios and consequently exclude a notable component of the dataset that do not meet the steady state conditions. As a consequence, only one period with wind speeds over 10 m s^−1^, *mP* 3 with 16 m s^−1^ winds, is included in the analysis, yet wind speeds up to 25 m s^−1^ were not uncommon. The cruise average, and median, was ~10 m s^−1^ with a standard deviation of 4 m s^−1^, whereas the median for the steady state cases was 7.5 m s^−1^ with similar standard deviation.

Overall during the study, the maximum observed ambient sea-salt submicron mass peaked at a concentration of 2.09 µg m^−3^, whereas nss-SO_4_ mass concentration reached only 1.12 µg m^−3^. Additionally, observations of 4 periods (see Figure S5) that just missed the criteria for steady-state classification show major sea-salt mass events (Event 1–4), where the sea-salt PM_1_ mass concentration exceeded 1.0 µg m^−3^. We acknowledge the nonlinearity between submicron particle mass and number concentration and studied the most stable cases (Event 1 and 4) in detail to resolve sea-salt and nss-SO_4_ number-size distribution particle concentrations and activated droplet concentrations. We observed 420 particles cm^−3^ in Event 1, which had a mean sea-salt mass concentration of 1.5 µg m^−3^ (~170 particles cm^−3^) with a nss-SO_4_ mean mass concentration of 0.13 µg m^−3^ (~250 cm^−3^), the highest sea-salt to nss-SO_4_ ratio. Event 2 had 460 particles cm^−3^ with a relative mean sea-salt and nss-SO_4_ mass concentration of 0.61 µg m^−3^ and 0.41 µg m^−3^, respectively. Event 3 had the largest particle concentration at 540 cm^−3^, with 0.9 µg m^−3^ of sea-salt and 0.3 µg m^−3^ of nss-SO_4_. Event 4 had the lowest number concentration at 140 particles cm^−3^, but a relatively high ratio of mean sea-salt mass to nss-SO_4_ with absolute concentrations of 0.92 µg m^−3^ (~110 cm^−3^) and 0.12 µg m^−3^ (~30 cm^−3^). We calculate that, in these high sea-salt:nss-SO_4_ mass events, sea-salt contributes 60–100% of the activated droplet concentration at a peak supersaturation <0.32% (see Table 2), exemplifying PMA, namely sea-salt, as a serious contributor to overall mass and CDNC.

These results illustrate that when realistic marine boundary layer cloud supersaturations are considered (e.g. ~0.2–0.3%, rather than 1%), on the whole SMA dominates the number fraction of activated cloud droplets at the higher end of realistic supersaturations. However, sea-salt can contribute significantly to the activated droplet concentration, challenging the seemingly accepted^37^ picture that sulphate is the only major contributor to marine CCN in most oceanic regions. More effort needs to be made to deconvolve the number fraction of sea-salt particles contributing to (relevant) CCN in the marine environment. This becomes especially important when considering that the susceptibility of the global cloud-radiation system is such that a 15–20% change in cloud properties (e.g. cloud extent and albedo) would be sufficient to counteract the radiative perturbation awarded by a doubling of CO_2_^38^ and we contend that, while SMA is often the controlling force for CDNC, at relevant supersaturation, sea salt can have a profound effect on activated droplets, susceptibility and thus, global albedo.

## Methods

### Cruise description

The PEGASO cruise, on board the Research Vessel *BIO Hesperides*, was organised by the Barcelona Institute of Marine Sciences. The vessel departed from Ushuaia on January 2^nd^, 2015 and returned to the same city on February 12^th^, 2015, after visiting the Antarctic Peninsula, the South Orkney Islands, and crossing the Scotia Sea to South Georgia. The ship course, marked in red in Fig. 1, facilitated on-board measurements of nascent aerosol to be taken in the Drake Passage, Scotia Sea and Northern Weddell Sea regions.

### *In-situ* meteorological and aerosol measurements

Standard meteorological parameters were monitored and recorded throughout the cruise, while aerosol measurements were taken from a laminar flow aerosol-dedicated sampling duct, 9 m in length and 1” outer diameter, with a PM_2.5_ cut-off at a flow rate of 5 L min^−1^, where overall residence time of particles in the main duct was 40 seconds prior to sampling by instruments. Instrumentation included a high-resolution time-of-flight aerosol mass spectrometer (*HR-ToF-AMS*) from Aerodyne Research Inc., a TSI model 3080 scanning-mobility particle sizer (*SMPS*) with TSI model 3010 condensation particle counter (*CPC*, flow 1 L min^−1^, range 0.01–0.5 µm), a single particle soot photometer (*SP2*) commercially available from Droplet Measurement Technologies, Inc. (DMT), and the continuous-flow streamwise thermal-gradient CCN counter (*CCNC*) commercially available from DMT^39,40^.

### Definition of steady state conditions for analysis

Aerosol spectral properties associated with air masses in the region were selected and analysed on the basis of periods of relatively stable or invariant meteorological and atmospheric composition characteristics. Air masses are associated with synoptic-scale meteorological systems and exhibit conserved meteorological parameters (e.g. equivalent potential temperature and total water mixing ratio) which are typically invariant within the air mass. Notable changes in the meteorological parameters at a particular location are normally more associated with large scale dynamics (e.g. frontal passage), representing a change in air mass, rather than small scale dynamical processes (e.g. entrainment and surface fluxes) that eventually transform the air mass with time. Similar to pseudo-steady-state meteorological parameters being characteristics of air mass origin, atmospheric composition variables (e.g. gases and aerosols) are also expected to possess pseudo-steady-state characteristics.

An arbitrary target of relative stability over durations of 8 hours was sought as the minimum requirement for selection and inclusion into the air mass characterisation database, although some inclusion of periods with stability over 4 hours occurred. In addition to relatively invariant meteorological parameters, size distribution parameters (e.g. modal size and number concentration) were required not to vary more than 20% from the mean. A similar requirement was afforded to aerosol chemical composition, particularly non-sea-salt sulphate (nss-SO_4_). Finally, a further selection criterion was that the back-trajectories had to indicate advection of the air over the same source region from start to end of the period.

### Size-segregated CCN particle analysis

The CCN Counter was set-up coupled to a differential mobility analyser (*DMA*) system to size segregate the aerosol^40,41^. The set-up sampled aerosol at 2 L min^−1^ through a nafion dryer. Eleven dry sizes were selected (20, 26, 33, 42, 54, 70, 90, 115, 148, 190, and 244 nm in mobility diameter) and each sampled for 60 seconds. After sizing the aerosol, the flow was split isokinetically between a CPC pulling 1 L min^−1^, and the CCNC pulling 0.5 L min^−1^ with a make-up flow of 0.5 L min^−1^ also added isokinetically. The time lag due to plumbing length was accounted for by rejecting the first 20 seconds of each size scan and averaging the remaining 40 seconds of data. Additionally, the first and second size scans were both 20 nm to allow CCNC temperatures to stabilize and reach a steady supersaturation. The CCNC cycled through five water supersaturations (0.08, 0.29, 0.47, 0.82, and 1.48%), such that each supersaturation level was held for the entire DMA size cycle. Critical dry diameters could then be obtained at a minimum time resolution of one hour. Critical diameters were later used in conjunction with the SMPS to find the average hourly total CCN at varying supersaturations. This is explained in more detail further on in this section.

### Resolving HR-ToF-AMS data

The size-resolved non-refractory chemical composition of submicron aerosol particles was measured with an Aerodyne High Resolution Time of Flight Aerosol Mass Spectrometer (HR-ToF-AMS, Aerodyne Research Inc., Billerica, MA). The instrument is described in DeCarlo *et al*.^42^. HR-ToF-AMS was routinely calibrated by ammonium nitrate according to the methods described by Jimenez *et al*.^43^ and Allan *et al*.^44^. Measurements were performed with a time resolution of 5 minutes, with a vaporizer temperature of about 600 °C. The composition-dependent collection efficiency^45^ was applied for the measurement periods discussed in this study. Sea-salt concentrations were measured following the method described in Ovadnevaite *et al*.^46^.

### Determining air mass origin and BC concentration

Air mass back-trajectories and satellite retrievals were obtained post-campaign. Air mass back-trajectories spanning 120 hours and arriving at the ship location at 100 m altitude obtained from HYSPLIT^27^, were used for origin guidance with consideration of their uncertainty after 30% of their 120 hour length. Black carbon mass was measured with the SP2 instrument, described in Stephens, *et al*.^47^ and commercially available at DMT, and applied along with trajectories to resolve the relative anthropogenic influence on the sampled aerosol.

### Satellite Chl-*a* data

Daily satellite sea surface chlorophyll concentration (Chl-*a*; mg m^−3^) and particulate backscattering (b_bp_ at 443 nm; m^−1^) version 3.0 data were taken from the Ocean Colour - Climate Change Initiative website (OC-CCI; http://www.esa-oceancolour-cci.org/). The OC-CCI project focuses on the estimation of the Ocean Colour Essential Climate Variables (ECVs), encompassing Chl-*a* concentration, remote-sensing reflectance in the visible domain (at SeaWiFS wavelengths), various other inherent optical properties as the b_bp_, and the diffuse attenuation coefficient at 490 nm. ESA-CCI products are the results of the merging of different sensors: SeaWiFS, MERIS, MODIS-Aqua, and VIIRS time-series.

OC-CCI version 3.0 Chl-*a* was estimated using a blending of OC5 algorithm^48,49^ and OC4v6-OCI algorithm^50^ as it performed better in the clearest oceanic waters (optical water classes 1 to 10), while b_bp_ was estimated with the global QAA algorithm^51^ (for more information see documents on http://www.esa-oceancolour-cci.org/). Both datasets were remapped at 0.1° resolution, enough to resolve the broader scales in the area of interest (the Patagonia Shelf). The dataset was from December 1^st^, 2014, until February 28^th^, 2015, for a total of 90 daily fields. Then, from the b_bp_, the phytoplankton carbon biomass was computed (C_phyto_; in terms of mg C m^−3^) following the original method of Behrenfeld *et al*.^52^ but considering the temporal variability of the b_bpNAP_ parameter, as described in Bellacicco *et al*.^53^. After that, multi-channel singular spectral analysis (M-SSA) was used to fill daily gaps^54^ in the Chl-*a* and derive C_phyto_ maps due to cloud cover or other environmental factors like sea ice. The method uses temporal as well as spatial correlation to fill the missing points (see Appendix A of Rinaldi *et al*.^55^ for additional details).

### CCN activation efficiency lines, percent contributions, kappa-value, and total concentration calculation

The size-segregated CCNC set-up probed the activation of aerosol in the size range of 20–244 nm as a function of supersaturation. The CCNC was calibrated with ammonium sulphate using the Aerosol Inorganics Model (the AIM activity coefficient Parameterization Model 3 [AP3]) method^40^. Following arguments made in Rose *et al*.^40^, since no sigmoidal shoulder appeared in the data, charge corrections were not deemed necessary. Each period was examined individually as follows: critical dry diameters (*D*_*c*_) for each supersaturation were resolved by plotting the fraction of activated CCN number to total condensation nuclei (CN) number concentration (N_CCN_/N_CN_) against dry particle mobility diameter and *D*_*c*_ was determined from the particle mobility diameter at which N_CCN_/N_CN_ = 0.5. Notionally, all particles larger than the critical diameter will freely activate into cloud droplets at the given supersaturation (*S*_*c*_). Taking values for *S*_*c*_ and plotting them against *D*_*c*_ gives a critical diameter activation curve which should be linear in log-log space for a given chemical composition (assuming that the solute mixture is non-volatile over the measurement and that the surface tension of the droplet is a constant value at any size). In this work, the CCN activation efficiency curves (or lines), shown for the different air mass types, result from the best linear fit to the log_10_(*S*_*c*_) *vs*. log_10_(*D*_*c*_) data (see Fig. 2).

The purpose of the activation curve is to compare the CCN activation capacity of ambient samples against known chemically uniform nuclei such as ammonium sulphate, sulphuric acid, ammonium methanesulphonate (NH_4_-MSA), and NaCl (proxy for sea-salt), to determine the relative activation efficiency, and also to elucidate the contribution of different sources or chemical components to the potential nuclei in the ambient air, explained below. For a given data point, the vertical location (or amplitude) on the plot quantifies the activation potential of environmental samples while the slope of the fitted activation curve based on the field measurements reveals a change of chemical composition with dry diameter or a change in the degree of internal *vs*. external mixing of aerosol populations in a common size range. Since the ambient aerosol consists of a mixture of chemical species, a perfect linear fit of the data in log_10_(*S*_*c*_) *vs*. log_10_(*D*_*c*_) space is not expected and the true slope at any measured data point may vary (reflecting an average chemical composition change with size; e.g. Fig. 2b).

*ĸ*-Köhler theory requires the use of a single hygroscopicity parameter, *κ*, to describe hygroscopic growth and/or CCN activation efficiency and is considered as an accepted standard in the field^28^. However, deviations in *κ* increase for decreasing particle size^56^ and adequate *κ*-values for the purpose of a CCN activity parameterisation as a function of dry diameter may show a substantial size-dependence in the sub-100 nm range, e.g. in the case of sulphuric acid (Ovadnevaite *et al*.^21^, Supplement). This study shows even greater deviations that may arise when the chemical composition of the aerosol is size dependent. For this reason, *κ*-values are listed with respect to the particle diameter (*D*_*p*_) for modal number-size distribution peaks (Tables 1 and 3).

**Table 3:** Log-normal parameter fits for number and volume size distributions.

	N		*ĸ*	V	
	Peak, nm	*δ*		Peak,nm	*δ*
*cAA 1*	24.4	1.3	0.48	—	—
	40.8	1.2	0.48	—	—
	101.5	1.5	0.50	130	1.4
	—	—	—	230	1.4
*cAA 2*	28.7	1.2	0.40	—	—
	46.2	1.2	0.46	—	—
	111.1	1.4	0.59	186	1.6
	—	—	—	480	1.1
*cAA 3*	44.1	1.2	0.43	—	—
	109.1	1.4	0.47	129.3	1.3
	—	—	—	214.2	1.4
*cAA 4*	40.6	1.3	0.38	46.7	1.2
	118.6	1.5	0.48	184.5	1.5
*mP 1*	40.5	1.3	0.34	49.6	1.3
	147	1.4	0.53	212	1.4
	—	—	—	455	1.2
*mP 2*	45.4	1.2	0.52	—	—
	109.5	1.5	0.61	211.4	1.7
	—	—	—	562.7	1.4
*mP 3*	27.1	1.2	0.51	—	—
	43.2	1.2	0.73	—	—
	114	1.5	1.43	160.4	1.4
	322.8	1.2	—	534.8	1.6
*mP 4*	39.5	1.3	0.42	49.8	1.3
	126.1	1.5	1.06	231.5	1.6
	304.2	1.5	—	589.3	1.4
*mP 5*	22.5	1.2	—	—	—
	45.9	1.3	0.47	53.3	1.5
	139.6	1.4	—	198	1.4
	—	—	—	456.6	1.2
*mT mod*.	43.2	1.3	0.52	—	—
*1*	74	1.3	0.58	94.1	1.3
	118	1.6	0.57	208.7	1.4
*mT 2*	46.8	1.3	0.55	57.3	1.3
	127.2	1.4	1.03	159.3	1.3
	343.7	1.2	—	391.6	1.4
*mT 3*	42.5	1.4	0.48	57	1.4
	136.3	1.4	—	177.6	1.3
	371	1.2	—	465.2	1.4

Particle size, modal spread (δ), and ĸ for fitted number—size distribution peaks shown on left. Fitted particle volume peaks and spread shown on right. Dashed lines represent the lack of more peaks in the volumetric distributions. Missing ĸ-values indicate that lognormally fitted peak diameters were outside of sizes resolved by the CCNC.

As CCN were measured for mono-dispersed particle mobility diameters, time trends of total ambient CCN numbers as a function of supersaturation were not measured by the CCNC. To resolve total N_CCN_ the hourly CCN critical dry diameters were applied to SMPS size-distribution data. It was assumed that, for pseudo-steady periods, critical dry diameters derived from the CCNC measurements would represent the run-away activation dry diameter – particles greater than this diameter would readily activate under a certain peak supersaturation level. Total N_CCN_ were derived from summing SMPS data collected for particle diameters greater than *D*_*c*_ as a function of supersaturation. In this study, N_CCN_ determined at 0.8% supersaturation can be considered a maximum value of marine CCN, (i) since derived marine N_CCN_ start reaching a maximum limit at supersaturations as low as 0.5% (see Fig. 2) and (ii) because the ambient marine cloud base will not typically exceed 0.8% supersaturation^32,33^.

In this paper, the inter-modal minimum is the critical diameter of free activation experienced by an aerosol population at cloud base. The inter-modal minimum is caused by a cloud peak supersaturation (*S*_*peak*_) which can be quantified by finding the corresponding *S*_*c*_ for the inter-modal, *D*_*c*,_ on the CCN ambient aerosol curves. As the ambient CCN curve is based on the average size-dependent physico-chemical aerosol population properties, the *D*_*c*_ for different chemical species at *S*_*peak*_ may be different from the ambient. PMA is considered here to be chemically represented by the theoretically derived *S*_*c*_*-D*_*c*_ curve of NaCl, and SMA is represented by the DON determined partially neutralised sulphate. This simplification is reasonable based on the small fractions of OM (excluding MSA) in either case compared with the inorganic components, which may otherwise affect the hygroscopic properties of the aerosol. Using the number-size distributions of the aerosol, and the *D*_*c*_ of SMA and PMA respectively, we can calculate the number contribution of the different aerosol types which would be considered activated into cloud droplets. First, we constrain the SMPS data by fitting a sea-salt distribution from the N. Atlantic (in the absence of ambient sea-salt distribution from the S. Ocean) from Ovadnevaite, *et al*.^57^. Using one spectral shape, from a wind speed of 6.3 m s^−1^ which is similar to the average wind speed witnessed for the cases (~7.5 m s^−1^), we scale the distribution based on mass calculations from the AMS. However, the AMS has a PM_1_ aerodynamic vacuum cut-off which must be converted to an equivalent mobility diameter^58^ for mass based scaling. The scaled sea-salt distributions are subtracted from the overall number size distributions with the remainder being assumed as the SMA number contribution. Using the *D*_*c*_ for either, the normalised number-size distributions of sea-salt (PMA) and SMA, respectively, are converted to absolute number and summed up for mobility diameters > *D*_*c*_. The total activated number is similarly the sum of the number-size distribution for sizes > inter-modal minimum. The percent contribution of PMA is determined as the fraction of activated PMA particles to the total activated number. Values derived for this calculation and the percent contributions can be found in Table 2 (*mP* 1 is omitted for lack of CCN data, and *mP* 4 is omitted due to large inter-modal minimum errors from number size distribution data instability). The inter-modal minimum calculation has an inherent uncertainty of ± 5%, which owes to the combination of a ±10% uncertainty in the SMPS size-binning which is somewhat reduced in the later fitting of the ambient data. The uncertainty of the CCN counter is ±0.02% for *S*_*peak*_ values. Due to the nature of the contribution calculation and the relatively steady nature of the PMA distribution, the maximum deviation in *D*_*c*_ or *S*_*peak*_ from either uncertainty would only result in a ±8% contribution change.

The model predictions for the critical dry diameter and critical supersaturation of selected solutes in aqueous droplets were carried out by a combination of the Aerosol Inorganic-Organic Mixtures Functional groups Activity Coefficients (AIOMFAC) model^59,60^ and application of the Köhler equation (Eq. 1), similar to the approach described in Ovadnevaite *et al*.^21^. However, since the relatively simple systems considered in the present study did not contain mixtures of organic and inorganic components, nor exhibit liquid-liquid phase separation, consideration of an evolving surface tension was not necessary.

1 $$S={a}_{w}\,\exp [\frac{4\,\sigma {M}_{w}}{RT{\rho }_{w}D}]$$

Here, *S* is the equilibrium water vapour saturation ratio of a spherical aqueous solution droplet of diameter *D* and mole-fraction-based water activity ${a}_{w}$ (both dependent on composition). exp[…] represents the exponential function with base *e*, *σ* is the effective liquid-air surface tension, *M*_*w*_ the molar mass of water, ${\rho }_{w}$ the liquid-state density of water at temperature *T*, and *R* the ideal gas constant. The AIOMFAC model predicts the molar composition and activities of all components, including ${a}_{w}$, as a function of input composition, thereby allowing the computation of water activities (i.e. bulk equilibrium RH) over a wide range of water contents. The molar solution composition can be converted into a droplet volume and sphere-equivalent diameter by using the molar masses and mass densities of the different mixture components. Additivity of volumes contributed by the individual components is assumed (assuming zero excess volume due to mixing). The density values used for the calculations are listed in Table S2. All calculations were performed assuming *T* = 293.15 K and a constant surface tension of *σ* = 72.75 mJ m^−2^, the value of the surface tension of pure water at *T*^61^. Critical supersaturation values (*S*_*c*_) were determined as the maximum values of *S* of the solute-specific Köhler curves computed with high numerical resolution in the high-water-activity range. The corresponding critical dry diameters, *D*_*c*_, were calculated from the water-free composition. The predicted *S*_*c*_ *vs. D*_*c*_ values are listed in Table S3.

Methanesulfonic acid (MSA) contains a chemical structural group that was not available in prior versions of AIOMFAC. Therefore, to provide AIOMFAC predictions for mixtures containing MSA and its sodium (Na-MSA) or ammonium (NH_4_-MSA) methanesulfonate salts, we introduce a new ionic subgroup, the methanesulfonate anion (CH_3_SO_3_^−^), to the list of ions considered by AIOMFAC. The relative van der Waals subgroup volume (*R*^*H*^) and surface area parameters (*Q*^*H*^) of the hydrated methanesulfonate anion were assumed to be equal to those of the hydrated sulphate ion; setting *R*^*H*^(CH_3_SO_3_^−^) = 3.34 and *Q*^*H*^(CH_3_SO_3_^−^) = 3.96^59^. The adjustable AIOMFAC middle-range parameters, describing the interactions of CH_3_SO_3_^−^ anions with the cations H^+^, Na^+^ and NH_4_^+^ in aqueous solutions, were determined by a weighted least-squares model-measurement optimization using the procedure described in Zuend, *et al*.^60^ based on experimental data sets by Covington, *et al*.^62^, Gregor, *et al*.^63^, Liu and Laskin^64^, and Peng and Chan^65^. A graphical comparison of the model predictions and the experimental data is shown in Figure S4. The fitted model parameters are listed in Table S4. We note that available experimental data on mixtures containing methanesulfonates as well as organic compounds is scarce; therefore, this extension of the AIOMFAC model is a system-specific approach applicable to aqueous, organic-free systems of the considered ions only.

### Estimation of aerosol exposure to biological activity

As an indicator of biological activity, Chl-*a* is an important proxy for marine productivity and biomass abundance. To represent the relative exposure of the sampled aerosol to biologically rich waters, HYSPLIT trajectory model outputs were overlaid onto satellite retrieved Chl-*a* concentrations in order to estimate the mean ocean Chl*-a* concentration traversed by the air mass back-trajectory path. The mean Chl*-a* concentration was calculated by first taking the accumulated concentration within pixels under the air mass back-trajectory path for the 72 hours prior to arriving at the ship, less the last three hours, and then taking the mean of all back-trajectory accumulated mean concentrations over the period. The last three hours are neglected to avoid introducing a bias into the dataset since significant secondary particle formation could not be expected to have occurred over that timescale before reaching the ship and given that the ship was likely to be located in the immediate vicinity of a bloom.

### Degree of neutralization (DON)

The degree of neutralization (DON_mol_), defined as the molar ratio of measured NH_4_^+^ to the quantity of NH_4_^+^ needed to fully neutralize the observed major inorganic anions in the aerosol, was calculated using the HR-ToF-AMS composition data for each of the periods selected, shown in Fig. 2 (Pie Chart). DON_mol_ ranges from 0 to 1 where DON_mol_ = 0 means no neutralization (i.e., sulphate and nitrate in dissolved acidic form) whereas a DON_mol_ = 1 means all measured SO_4_ and NO_3_ exists as ammonium sulphate and ammonium nitrate components, respectively. The DON_mol_ value is calculated by ionic balance as

2 $${{\rm{DON}}}_{{\rm{mol}}}=\frac{{n}_{{{\rm{NH}}}_{4}^{+}}}{2\times {n}_{{{\rm{SO}}}_{4}^{2-}}+{n}_{{{\rm{NO}}}_{3}^{-}}},$$

where ${n}_{{{\rm{NH}}}_{4}^{+}}$, ${n}_{{{\rm{SO}}}_{4}^{2-}}$ and ${n}_{{{\rm{NO}}}_{3}^{-}}$ denote the molar amounts of the indicated ionic species determined for a specific sample. The measured nitrate amounts were small compared to both the sulphate and ammonium molar amounts. The ${{\rm{DON}}}_{{\rm{mol}}}$ determined from the field data were then used in conjunction with predictions from the AIOMFAC model for aqueous particles consisting of ammonium sulphate mixed with sulphuric acid, providing a comparison of the CCN properties at the same ${{\rm{DON}}}_{{\rm{mol}}}$ as well as over the full range of ${{\rm{DON}}}_{{\rm{mol}}}$ possible.

## Electronic supplementary material

Supplementary Information
