# Author Correction: Summertime Primary and Secondary Contributions to Southern Ocean Cloud Condensation Nuclei

**DOI:** 10.1038/s41598-019-46788-3

**Published:** 2019-07-18

**Authors:** Kirsten N. Fossum, Jurgita Ovadnevaite, Darius Ceburnis, Manuel Dall’Osto, Salvatore Marullo, Marco Bellacicco, Rafel Simó, Dantong Liu, Michael Flynn, Andreas Zuend, Colin O’Dowd

**Affiliations:** 1 School of Physics, Ryan Institute’s Centre for Climate & Air Pollution Studies, and Marine Renewable Energy Ireland, National University of Ireland Galway, University Road, Galway, H91 CF50 Ireland; 2 Institut de Ciències del Mar (CSIC), Barcelona, Catalonia Spain; 3 Agenzia nazionale per le nuove tecnologie, l’energia e lo sviluppo económico sostenibile, ENEA — Centro Ricerche Frascati, Frascati, Italy; 4 Institute of Atmospheric Sciences and Climate (ISAC), Rome, Italy; 5 Sorbonne Université, CNRS, Laboratoire d’Océanographie de Villefranche, LOV, F-06230 Villefranchesur-Mer, France; 6 Centre for Atmospheric Sciences, School of Earth and Environmental Sciences, University of Manchester, Manchester, M13 9PL UK; 7 Department of Atmospheric and Oceanic Sciences, McGill University, Montreal, Quebec Canada

Correction to: *Scientific Reports* 10.1038/s41598-018-32047-4, published online 14 September 2018

The original version of this Article contained an error in Affiliation 1, which was incorrectly given as ‘School of Physics, Ryan Institute’s Centre for Climate & Pollution Studies, and Marine Renewable Energy Ireland, National University of Ireland Galway, University Road, Galway, H91 CF50, Ireland’. The correct affiliation is listed below:

School of Physics, Ryan Institute’s Centre for Climate & Air Pollution Studies, and Marine Renewable Energy Ireland, National University of Ireland Galway, University Road, Galway, H91 CF50, Ireland.

This error has now been corrected in the HTML and PDF versions of the Article, and in the accompanying Supplementary Information file.

