# Supplementary material for: Summertime Primary and Secondary Contributions to Southern Ocean Cloud Condensation Nuclei
Source: Sci Rep. 2018 Sep 14;8:13844. doi: 10.1038/s41598-018-32047-4 (PMC6138724; doi:10.1038/s41598-018-32047-4)
Supplement: Supplementary file 1 — Supplementary Information [file 41598_2018_32047_MOESM1_ESM.pdf]

Supplementary Information

Summertime Primary and Secondary Contributions to Southern Ocean Cloud Condensation Nuclei

Kirsten N. Fossum[1*], Jurgita Ovadnevaite[1], Darius Ceburnis[1], Manuel Dall'Osto[2], Salvatore Marullo[3,4], Marco Bellacicco[4, 5], Rafel Simó[2], Dantong Liu[6], Michael Flynn[6], Andreas Zuend[7], Colin O'Dowd[1*]

[1]School of Physics, Ryan Institute's Centre for Climate & Air Pollution Studies, and Marine Renewable Energy Ireland, National University of Ireland Galway. University Road, Galway. H91 CF50, Ireland.

[2]Institut de Ciències del Mar (CSIC), Barcelona, Catalonia, Spain.

[3]Agenzia nazionale per le nuove tecnologie, l'energia e lo sviluppo economico sostenibile, ENEA — Centro Ricerche Frascati, Frascati, Italy.

[4]Institute of Atmospheric Sciences and Climate (ISAC), Rome, Italy.

[5]Sorbonne Université, CNRS Laboratoire d'Océanographie de Villefranche LOV, F-06230 Villefranche-sur-Mer France.

[6]Centre for Atmospheric Sciences, School of Earth and Environmental Sciences, University of Manchester, Manchester M13 9PL, UK.

[7]Department of Atmospheric and Oceanic Sciences, McGill University, Montreal, Quebec, Canada.

Table S1. Inter-modal minima points and corresponding parameters listed according to their occurrence: inter-modal diameter, number of particles at the inter-modal diameter, critical supersaturation corresponding to inter-modal minimum and number of particles above inter-modal minimum diameter.

	$D_{int\text{-}mod\ min}$, nm	N at $D_{int\text{-}mod\ min}$, cm^{-3}	S_c, %	$N_{>int\text{-}mod\ min}$, cm^{-3}
cAA 1	55.3	3.8	0.41	212.3
cAA 2	61.0	2.7	0.35	165.4
cAA 3	54.0	2.5	0.45	208.5
cAA 4	65.0	3.0	0.34	175.9
re. *mP* 1	78.2	2.2	-	142.0
mP 2	63.9	4.4	0.31	221.3
mP 3	68.8	1.5	0.20	65.3
mP 4	82.1	3.7	0.18	191.0
mP 5	81.7	5.0	0.19	263.6
mT mod. 1	-	-	-	-
mT 2	78.7	6.3	0.20	253.1
mT 3	83.4	4.8	0.17	207.0
Case *cAA*	55.9	3.2	0.40	201.9
Case *mP*	81.8	3.4	0.19	169.8

Table S2. Liquid- and solid-state densities of pure components at $T \approx 20$ °C (and ~10^5 Pa) used for the AIOMFAC-based calculations of critical CCN properties.

Chemical compound (physical state)	Density ρ (kg m^{-3})
H_2O (liquid)	997
H_2SO_4 (liquid)	1860 [a]
$(NH_4)_2SO_4$ (liquid)	1550 [b]
$(NH_4)_2SO_4$ (solid)	1770 [b]
NH_4HSO_4 (liquid)	1730 [b]
NH_4HSO_4 (solid)	1780 [b]
Methanesulfonic acid (MSA), (liquid)	1650 [c]
Sodium methanesulfonate (Na-MSA), (liquid)	2190 [d]
Ammonium methanesulfonate (NH_4-MSA), (liquid)	1440 [e]

[a] Value obtained using the temperature-dependent parameterisation by Myhre, et al. [66].
[b] Solid- (crystalline) and liquid-state values from Clegg and Wexler [67].
[c] Calculated based on apparent molar volume data of 7-molal aqueous solution by Teng and Lenzi [68].
[d] Calculated based on apparent molar volume data by Tamaki, et al. [69].
[e] Determined using Eq. (1) of Kosova, et al. [70].

Table S3. Critical supersaturation S_c (%) listed for a selection of corresponding critical dry diameters (D_c) from 20 to 200 nm predicted by the AIOMFAC model with Köhler theory. DON_{mol} indicates the degree of sulphate neutralization by ammonia (Eq. 1, main text) for solute mixtures containing ammonium sulphate and sulphuric acid at input; all data for T = 293.15 K and σ = 72.75 mJ m^{-2}.

Mixture		Dry diameter (nm)														
Solutes (at input)	DON_{mol}	20	25	30	35	40	45	50	60	70	80	100	120	140	160	200
H_2SO_4	0.00	1.667	1.191	0.902	0.713	0.580	0.484	0.411	0.309	0.243	0.197	0.139	0.104	0.082	0.066	0.047
$(NH_4)_2SO_4$, H_2SO_4	0.10	1.708	1.218	0.922	0.727	0.592	0.493	0.419	0.315	0.248	0.201	0.142	0.106	0.083	0.068	0.048
$(NH_4)_2SO_4$, H_2SO_4	0.20	1.747	1.243	0.940	0.741	0.603	0.502	0.426	0.320	0.252	0.204	0.144	0.108	0.085	0.069	0.048
$(NH_4)_2SO_4$, H_2SO_4	0.30	1.782	1.267	0.956	0.754	0.612	0.510	0.433	0.325	0.255	0.207	0.146	0.110	0.086	0.070	0.049
$(NH_4)_2SO_4$, H_2SO_4	0.40	1.814	1.287	0.971	0.764	0.621	0.517	0.438	0.330	0.259	0.210	0.148	0.111	0.087	0.071	0.050
$(NH_4)_2SO_4$, H_2SO_4	0.50	1.841	1.304	0.983	0.773	0.628	0.522	0.443	0.333	0.262	0.212	0.150	0.112	0.088	0.072	0.051
$(NH_4)_2SO_4$, H_2SO_4	0.60	1.861	1.316	0.991	0.779	0.632	0.526	0.446	0.335	0.263	0.214	0.151	0.113	0.089	0.072	0.051
$(NH_4)_2SO_4$, H_2SO_4	0.70	1.876	1.325	0.996	0.783	0.635	0.528	0.448	0.337	0.265	0.215	0.152	0.114	0.090	0.073	0.052
$(NH_4)_2SO_4$, H_2SO_4	0.75	1.882	1.328	0.998	0.784	0.636	0.529	0.449	0.337	0.265	0.215	0.152	0.114	0.090	0.073	0.052
$(NH_4)_2SO_4$	1.00	1.905	1.340	1.005	0.788	0.639	0.532	0.451	0.339	0.267	0.217	0.154	0.116	0.091	0.074	0.053
NaCl	N/A	1.231	0.878	0.666	0.527	0.430	0.360	0.307	0.233	0.184	0.151	0.107	0.082	0.065	0.053	0.038
MSA	N/A	1.772	1.271	0.968	0.768	0.629	0.527	0.449	0.342	0.271	0.221	0.158	0.120	0.095	0.078	0.056
Na-MSA	N/A	1.738	1.240	0.941	0.745	0.609	0.510	0.435	0.330	0.261	0.213	0.152	0.116	0.092	0.075	0.053
NH_4-MSA	N/A	2.107	1.505	1.142	0.905	0.739	0.618	0.527	0.400	0.316	0.258	0.184	0.140	0.111	0.091	0.065

Table S4. Fitted binary cation—anion AIOMFAC middle-range interaction parameters[60] for aqueous solutions of MSA, Na-MSA, and NH_4-MSA; determined for $T \approx 298.15$ K.

c	a	$b_{c,a}^{(1)}$ (kg mol^{-1})	$b_{c,a}^{(2)}$ (kg mol^{-1})	$b_{c,a}^{(3)}$ (kg$^{1/2}$ mol$^{-1/2}$)	$c_{c,a}^{(1)}$ (kg^2 mol^{-2})	$c_{c,a}^{(2)}$ (kg$^{1/2}$ mol$^{-1/2}$)
H^+	$CH_3SO_3^-$	4.67900×10^{-2}	4.56220×10^{-1}	2.40000×10^{-1}	-1.70295×10^{-1}	1.42818×10^{-1}
Na^+	$CH_3SO_3^-$	6.90732×10^{-3}	3.16640×10^{-1}	3.25673×10^{-1}	-4.38075×10^{-1}	2.10970×10^{0}
NH_4^+	$CH_3SO_3^-$	2.33752×10^{-2}	2.23470×10^{-2}	2.20000×10^{-1}	2.47270×10^{-2}	8.26574×10^{-1}

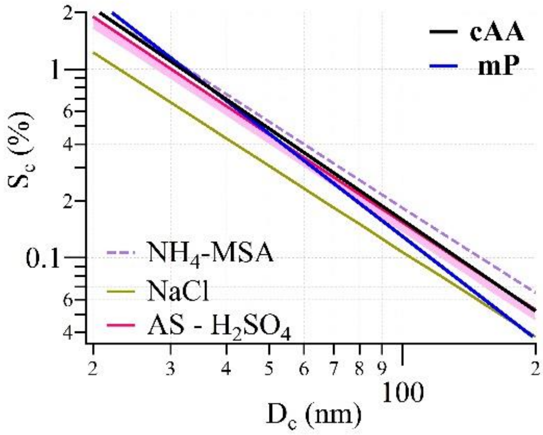

Figure S1. CCN activation efficiency as a function of critical supersaturation and diameter. Two merged cases, *cAA* and *mP*, shown in black and blue respectively. AIOMFAC model-predicted values of NaCl in olive brown, sulphate varying degree of neutralization in pink covering the DON_{mol} range from H_2SO_4 to $(NH_4)_2SO_4$ (dark pink), and ammonia-neutralised MSA (i.e. NH_4-MSA salt) in dashed purple.

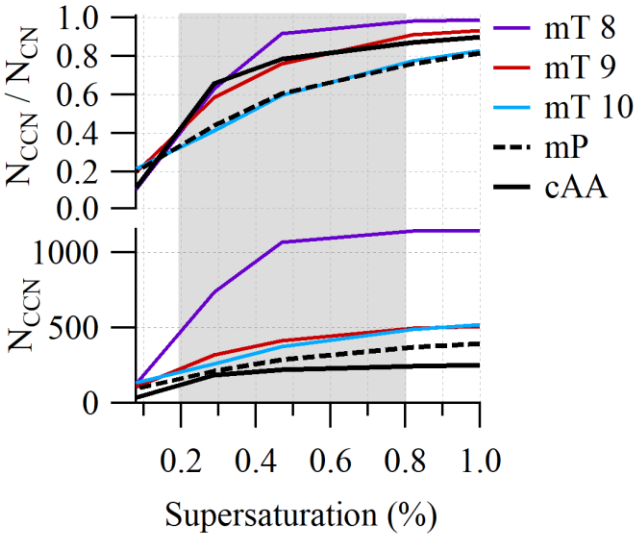

Figure S2. On top, the ratio of CCN to all particles greater than 20 nm, listed by air mass source region. On bottom, the total number of CCN for varying supersaturation. Shaded range of supersaturations represent typical values for marine stratocumulus clouds.

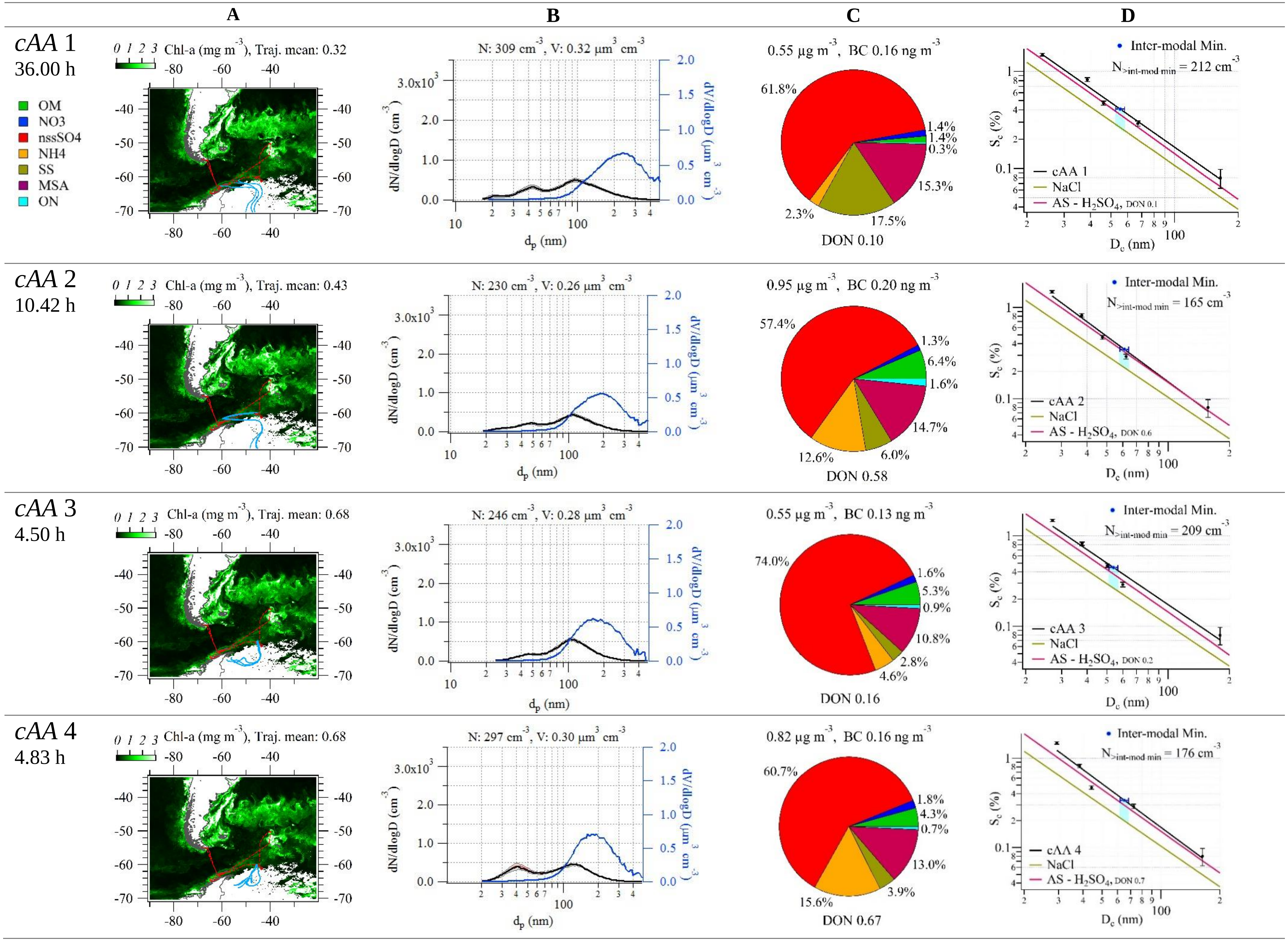

A
B
C
D
cAA 1
36.00 h
OM
NO3
nssSO4
NH4
SS
MSA
ON
0 1 2 3 Chl-a (mg m-3), Traj. mean: 0.32
N: 309 cm-3, V: 0.32 µm3 cm-3
dN/dlogD (cm-3)
dV/dlogD (µm3 cm-3)
dp (nm)
0.55 µg m-3, BC 0.16 ng m-3
61.8%
1.4%
1.4%
0.3%
15.3%
17.5%
2.3%
DON 0.10
Inter-modal Min.
N>int-mod min = 212 cm-3
Sc (%)
Dc (nm)
cAA 1
NaCl
AS - H2SO4, DON 0.1
cAA 2
10.42 h
0 1 2 3 Chl-a (mg m-3), Traj. mean: 0.43
N: 230 cm-3, V: 0.26 µm3 cm-3
0.95 µg m-3, BC 0.20 ng m-3
57.4%
1.3%
6.4%
1.6%
14.7%
6.0%
12.6%
DON 0.58
Inter-modal Min.
N>int-mod min = 165 cm-3
cAA 2
NaCl
AS - H2SO4, DON 0.6
cAA 3
4.50 h
0 1 2 3 Chl-a (mg m-3), Traj. mean: 0.68
N: 246 cm-3, V: 0.28 µm3 cm-3
0.55 µg m-3, BC 0.13 ng m-3
74.0%
1.6%
5.3%
0.9%
10.8%
2.8%
4.6%
DON 0.16
Inter-modal Min.
N>int-mod min = 209 cm-3
cAA 3
NaCl
AS - H2SO4, DON 0.2
cAA 4
4.83 h
0 1 2 3 Chl-a (mg m-3), Traj. mean: 0.68
N: 297 cm-3, V: 0.30 µm3 cm-3
0.82 µg m-3, BC 0.16 ng m-3
60.7%
1.8%
4.3%
0.7%
13.0%
3.9%
15.6%
DON 0.67
Inter-modal Min.
N>int-mod min = 176 cm-3
cAA 4
NaCl
AS - H2SO4, DON 0.7

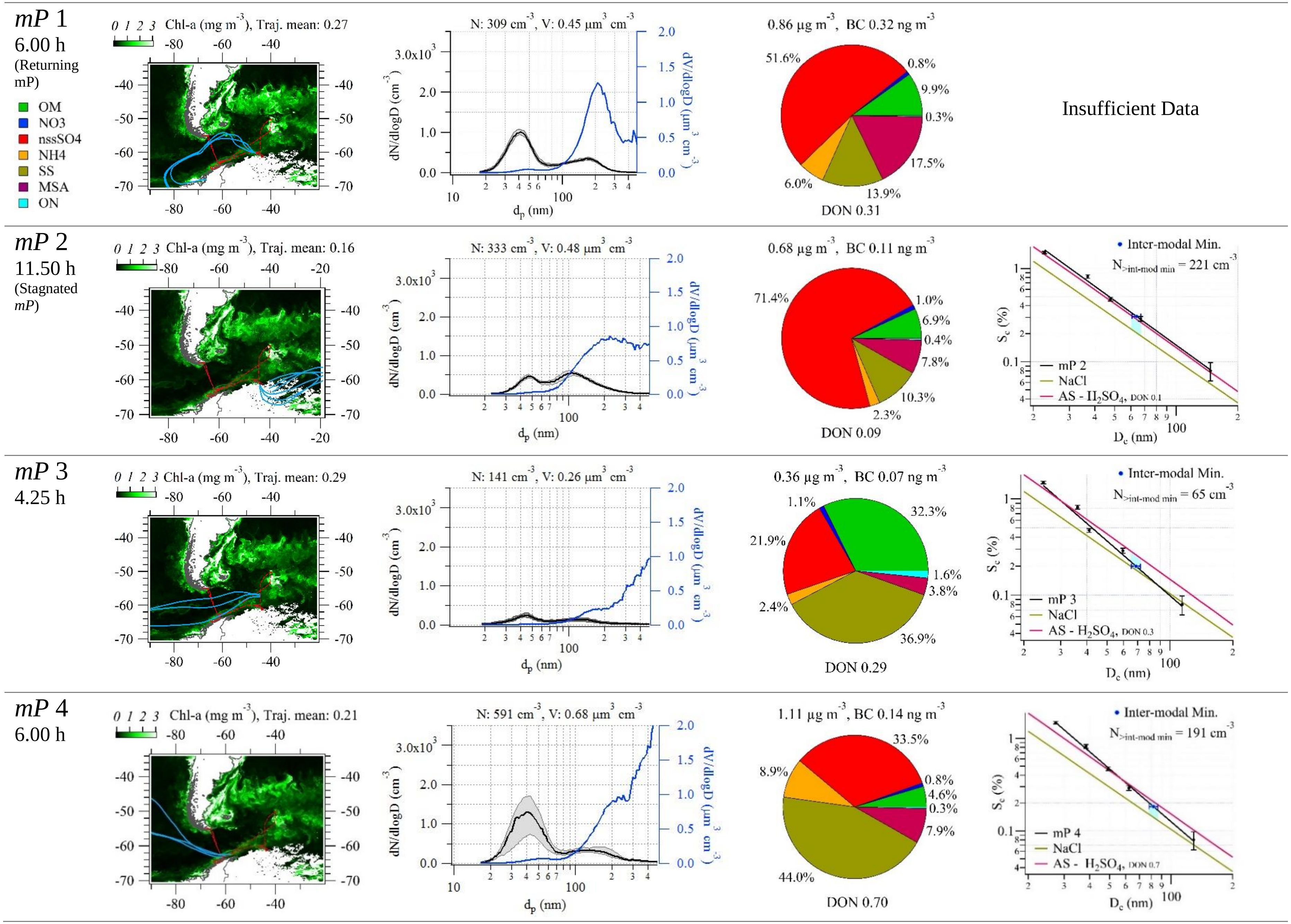
mP 1
6.00 h
(Returning mP)
OM
NO3
nssSO4
NH4
SS
MSA
ON
Chl-a (mg m-3), Traj. mean: 0.27
N: 309 cm-3, V: 0.45 µm3 cm-3
0.86 µg m-3, BC 0.32 ng m-3
51.6%
0.8%
9.9%
0.3%
17.5%
13.9%
6.0%
DON 0.31
Insufficient Data
mP 2
11.50 h
(Stagnated mP)
Chl-a (mg m-3), Traj. mean: 0.16
N: 333 cm-3, V: 0.48 µm3 cm-3
0.68 µg m-3, BC 0.11 ng m-3
71.4%
1.0%
6.9%
0.4%
7.8%
10.3%
2.3%
DON 0.09
Inter-modal Min.
N>int-mod min = 221 cm-3
mP 2
NaCl
AS - H2SO4, DON 0.1
mP 3
4.25 h
Chl-a (mg m-3), Traj. mean: 0.29
N: 141 cm-3, V: 0.26 µm3 cm-3
0.36 µg m-3, BC 0.07 ng m-3
1.1%
32.3%
21.9%
1.6%
3.8%
2.4%
36.9%
DON 0.29
Inter-modal Min.
N>int-mod min = 65 cm-3
mP 3
NaCl
AS - H2SO4, DON 0.3
mP 4
6.00 h
Chl-a (mg m-3), Traj. mean: 0.21
N: 591 cm-3, V: 0.68 µm3 cm-3
1.11 µg m-3, BC 0.14 ng m-3
33.5%
8.9%
0.8%
4.6%
0.3%
7.9%
44.0%
DON 0.70
Inter-modal Min.
N>int-mod min = 191 cm-3
mP 4
NaCl
AS - H2SO4, DON 0.7
dN/dlogD (cm-3)
dV/dlogD (µm3 cm-3)
dp (nm)
Sc (%)
Dc (nm)

mP 5
7.00 h

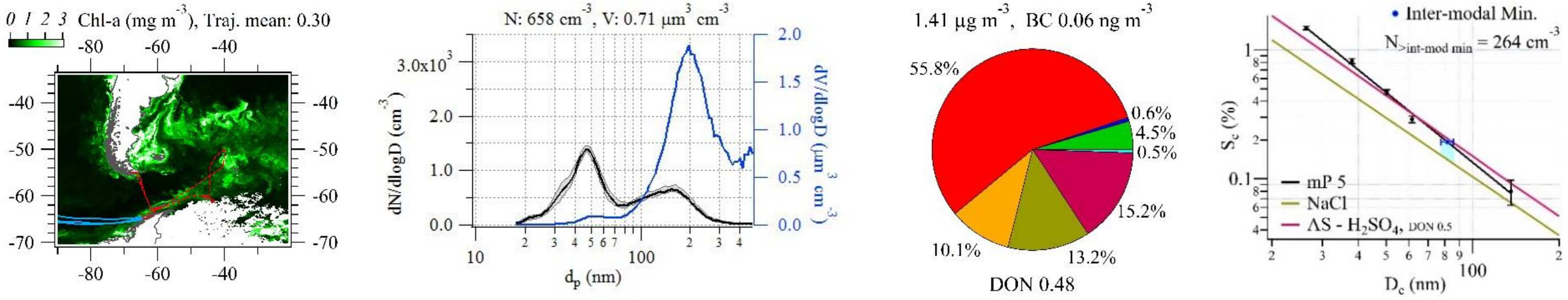

mT 1
8.50 h
(Modified mT)

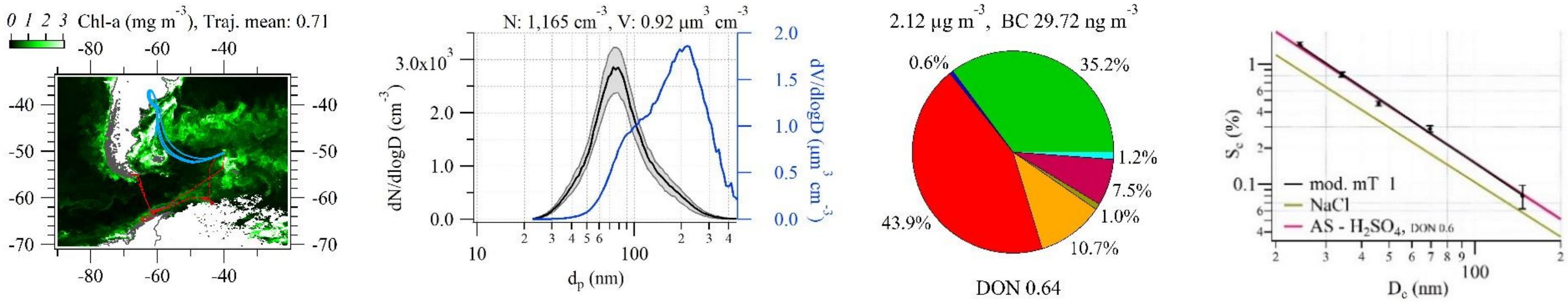

mT 2
5.92 h

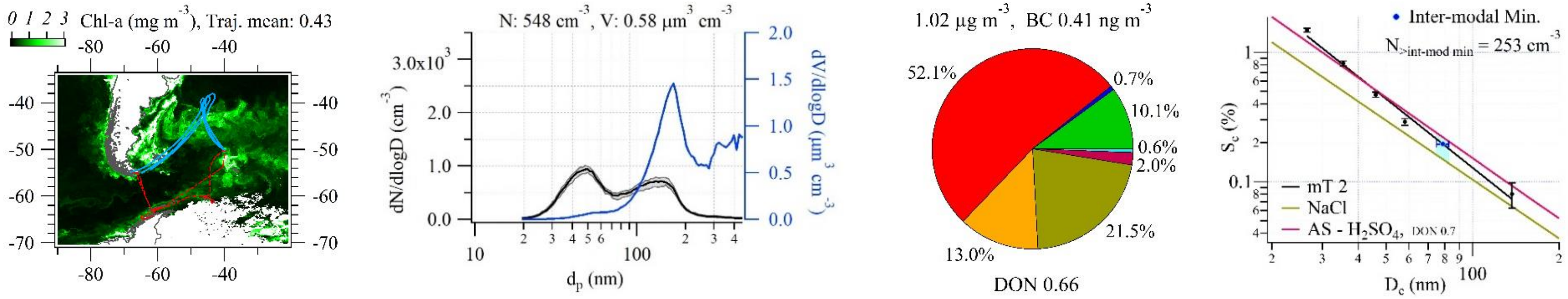

mT 3
6.00 h

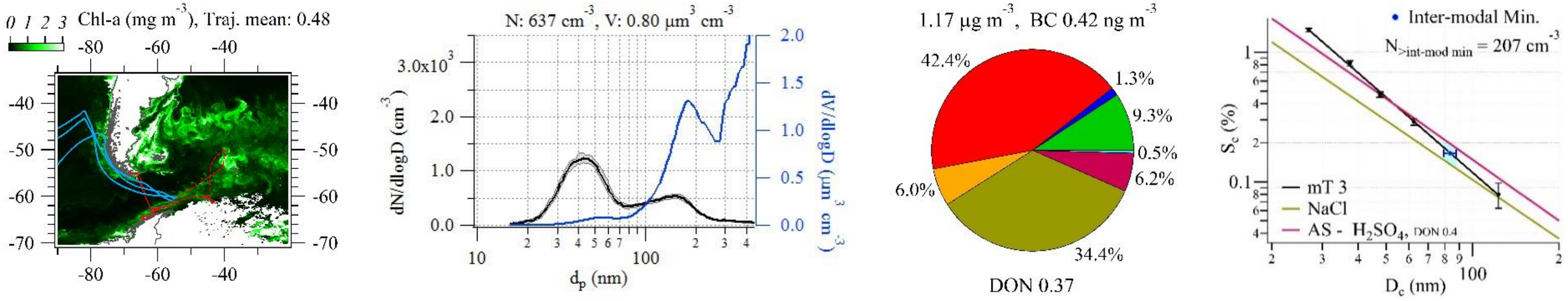

Figure S3. Aerosol chemical and physical characterization, each presented period listed in chronological order by air mass. Each period has 5 columns, descriptor, A, B, C, and D. The descriptor lists period number followed by any air mass modification then the steady-state duration of the period. [A] Chl-*a* satellite daily retrievals with overlaid AMBT extracted from HYSPLIT [27,71]. In dotted red, PEGASO cruise ship path. In blue, 120 hr back trajectory ending 100m AGL directly above the ship location. There are three trajectories, representing the air mass origin at the start, middle, and end of the period. Chl-*a* satellite retrievals are at 0.1º x 0.1º resolution, where the darkest green represents close to 0 mg m^{-3}, and nearly white represents 3 mg m^{-3} average Chl-*a* in that pixel. Chl-*a* scale on the top left and the average Chl-*a* concentration during the period under the trajectories on the top right corner. [B] Particle number (black) and volume (blue) size distributions: number distribution variance during the period is shown in grey. On top are the total average number of particles [cm^{-3}] (N), and total average particulate volume [μm^3 cm^{-3}] (V). [C] Pie chart of chemical mass fractions (OM: organic matter, SS: sea-salt, ON: organic nitrogen). On top, submicron particle mass [μg m^{-3}] followed by black carbon mass [ng m^{-3}] (BC). On bottom, degree of neutralization (DON) where 1 means that all sulphate and nitrate exist as ammonium sulphate and ammonium nitrate [58]. [D] CCN activation efficiency curves. Graph shows critical supersaturation against critical dry diameter; the slope derived from the measurements during the period is shown in black (supersaturation uncertainty presented by vertical error bars), and the predicted slopes of partially neutralized (based on DON) sulphuric acid from the AIOMFAC model shown in red while the NaCl line shown in olive brown predicted by AP3 model [40]. The inter-modal minimum point, extracted from the log-normal fit of number-size-distributions, shown in blue with ±5% size error. At top, the number of particles larger than the inter-modal minimum.

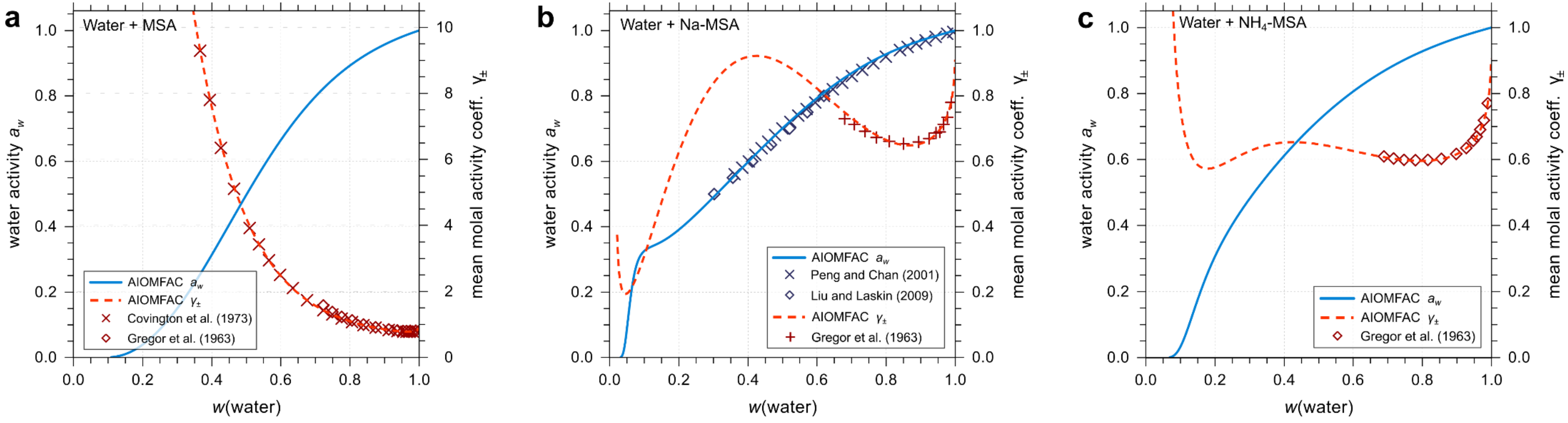

Figure S4. Comparison of experimental data and AIOMFAC predictions for water activity (left y-axis) and the mean molal ion activity coefficient (right y-axis) of the binary aqueous systems used to fit the model parameters at $T \approx 298$ K. The solutes are MSA (**a**), Na-MSA (**b**) and NH_4-MSA (**c**).

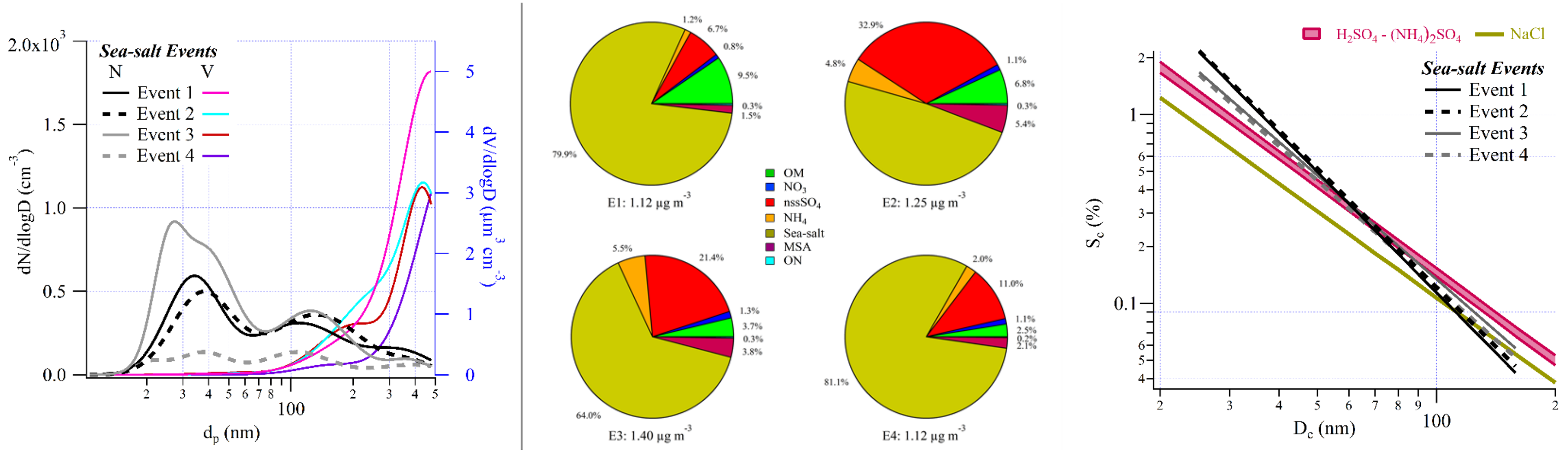

Figure S5. Sea-salt Event analysis shown for SMPS, HR-ToF-AMS, and CCNC data shown. The left panel showing number (N) and volume (V) size distributions of Event 1 -4 (E1-4). Black and grey solid and dashed lines represent the number-size distributions while the coloured solid lines represent the volume distributions. The middle panel shows AMS chemical fractions of all four events with total mass concentration at the bottom of each pie-chart, respectively. The right panel shows CCN activation efficiency curves of sea-salt events with critical supersaturation plotted against critical dry diameter; the slopes derived from the measurements during the periods are shown. The AIOMFAC model predicted NaCl line, in olive brown, and the sulphuric acid to neutralized sulphate (H_2SO_4 – $(NH_4)_2SO_4$) range are shown.

i)

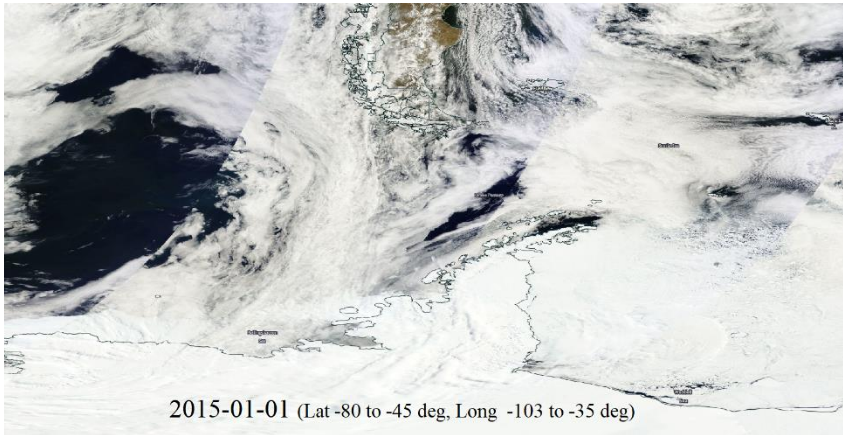

ii)

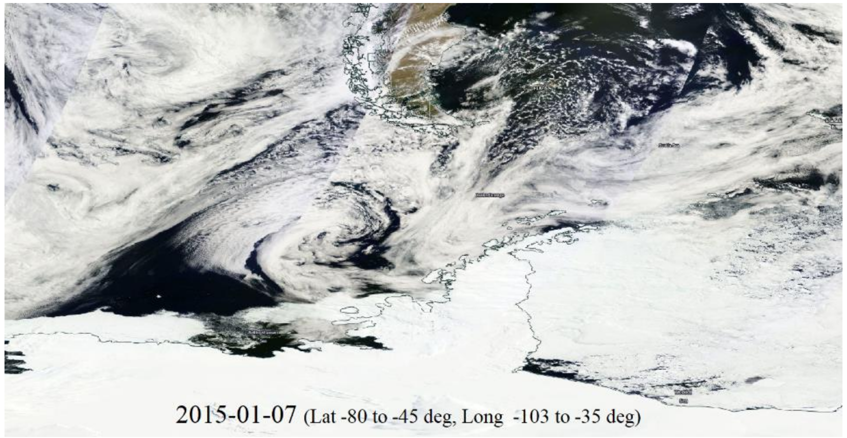

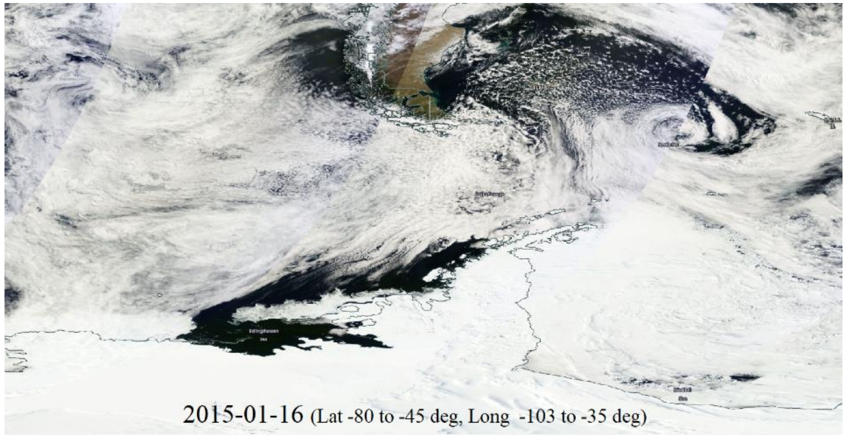

iii)

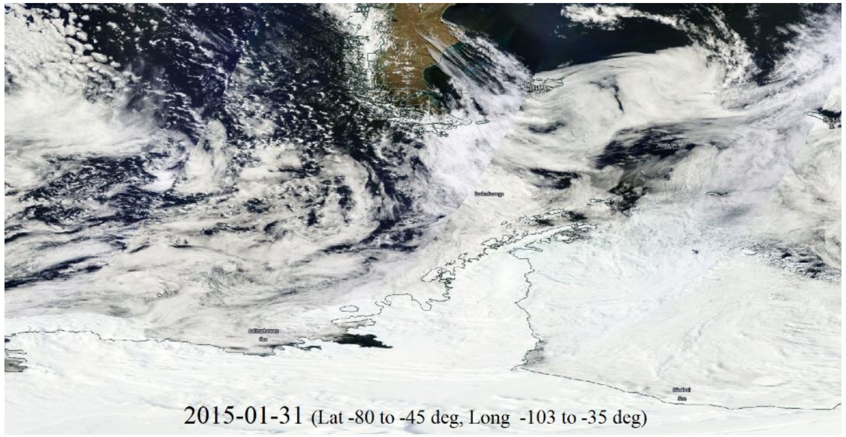

iv)

Figure S6. Satellite images taken from NASA Worldview[72] (https://worldview.earthdata.nasa.gov/) for four separate days in January 2015; (i) Jan 1st, (ii) Jan 7th, (iii) Jan 16th, and (iv) Jan 31st. Images show daily resolution of the cloudiness in the region and are representative of the cloud cover occurring for the extent of the PEGASO cruise. Each image is at ~5 km resolution and shows a composite of each day at latitudes from -80 to -45 degrees and longitudes from -103 to -35 degrees.

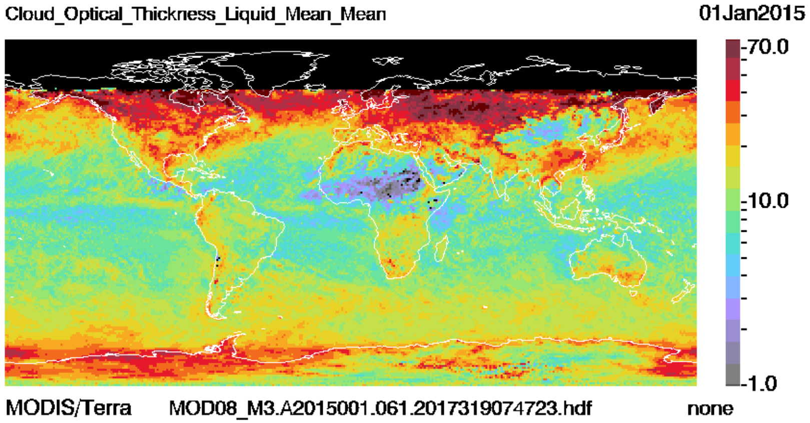

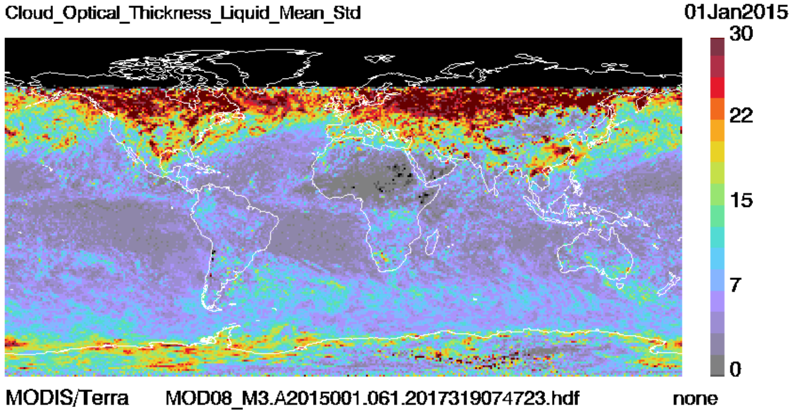

Figure S7. Satellite monthly mean composites of liquid cloud optical thickness (left) and the monthly standard deviation (right) globally. Images taken of January 2015 from MODIS L3 monthly collection 6.1, [https://modis-atmos.gsfc.nasa.gov/images/l3-monthly-browse][73].

References

[66] Myhre, C. E. L., Nielsen, C. J. & Saastad, O. W. Density and Surface Tension of Aqueous H2SO4 at Low Temperature. *Journal of Chemical & Engineering Data* **43**, 617-622, doi:10.1021/je980013g (1998).

[67] Clegg, S. L. & Wexler, A. S. Densities and Apparent Molar Volumes of Atmospherically Important Electrolyte Solutions. 1. The Solutes H2SO4, HNO3, HCl, Na2SO4, NaNO3, NaCl, (NH4)2SO4, NH4NO3, and NH4Cl from 0 to 50 °C, Including Extrapolations to Very Low Temperature and to the Pure Liquid State, and NaHSO4, NaOH, and NH3 at 25 °C. *The Journal of Physical Chemistry A* **115**, 3393–3460, https://doi.org/10.1021/jp108992a(2011).

[68] Teng, T. T. & Lenzi, F. Methanesulfonic and trichloroacetic acids. Densities of aqueous solutions at 20.deg., 25.deg., and 35.deg. *Journal of Chemical & Engineering Data* **20**, 432–434, https://doi.org/10.1021/je60067a008 (1975).

[69] Tamaki, K., Ohara, Yk, Inabe, M., Mori, T. & Numata, F. The Physicochemical Properties of Aqueous Solutions of Sodium Alkanesulfonates. Apparent Molar Volumes, Viscosity B Coefficients, Heats of Solution, and Surface Tensions. *Bulletin of the Chemical Society of Japan* **56**, 1930–1934, https://doi.org/10.1246/bcsj.56.1930 (1983).

[70] Kosova, D. A., Navalayeu, T. I., Maksimov, A. I., Babkina, T. S. & Uspenskaya, I. A. Experimental investigation of the solid – Liquid phase equilibria in the water – Ammonium methanesulfonate and in the water – Sodium methanesulfonate systems. *Fluid Phase Equilibria* **443**, 23–31, https://doi.org/10.1016/j.fluid.2017.04.006 (2017).

[71] Rolph, G. D. *Real-time Environmental Applications and Display sYstem (READY) Website* http://www.ready.noaa.gov/index.php (2016).

[72] NASA. In *Earth Science Data and Information System (ESDIS) Project* (Goddard Space Flight Center, Maryland, USA, https://worldview.earthdata.nasa.gov/ (2018).

[73] NASA. In *Collection 6*.1(ed MOD08_M3.A2015001.061.2017319074723.hdf) (Goddard Space Flight Center, Greenbelt, Maryland, USA, https://modis-images.gsfc.nasa.gov/MOD08_M3/_BROWSE_FIXEDSCALE/2015_01/C061/LL/browse_main_m7 (2017).
